# Potential Biomarkers for the Earlier Diagnosis of Kidney and Liver Damage in Acute Intermittent Porphyria

**DOI:** 10.3390/life14010019

**Published:** 2023-12-21

**Authors:** Elin Storjord, Staffan Wahlin, Bård Ove Karlsen, Randolf I. Hardersen, Amy K. Dickey, Judith K. Ludviksen, Ole-Lars Brekke

**Affiliations:** 1 Department of Laboratory Medicine, Nordland Hospital Trust, 8092 Bodø, Norway; bard.ove.karlsen@nordlandssykehuset.no (B.O.K.); ole.lars.brekke@nordlandssykehuset.no (O.-L.B.); 2 Hepatology Division, Department of Upper GI Diseases, Porphyria Centre Sweden, Karolinska Institute and Karolinska University Hospital, 14186 Stockholm, Sweden; staffan.wahlin@ki.se; 3 Research Laboratory, Nordland Hospital Trust, 8092 Bodø, Norway; jukrey@gmail.com; 4 Department of Nephrology, Nordland Hospital Trust, 8092 Bodø, Norway; 5 Department of Clinical Medicine, UiT-The Arctic University of Norway, 9019 Tromsø, Norway; 6 Department of Medicine, Massachusetts General Hospital, Boston, MA 02114, USA; adickey@mgh.harvard.edu; 7 Harvard Medical School, Boston, MA 02115, USA

**Keywords:** acute intermittent porphyria, porphyria, biomarker, α-glutathione S-transferase, cytokines, delta-aminolevulinic acid, fatty acid-binding protein-1, inflammation, kidney injury molecule-1, porphobilinogen

## Abstract

Acute intermittent porphyria (AIP) is an inherited metabolic disorder associated with complications including kidney failure and hepatocellular carcinoma, probably caused by elevations in the porphyrin precursors porphobilinogen (PBG) and delta-aminolevulinic acid (ALA). This study explored differences in modern biomarkers for renal and hepatic damage between AIP patients and controls. Urine PBG testing, kidney injury panels, and liver injury panels, including both routine and modern biomarkers, were performed on plasma and urine samples from AIP cases and matched controls (50 and 48 matched pairs, respectively). Regarding the participants’ plasma, the AIP cases had elevated kidney injury marker-1 (KIM-1, *p* = 0.0002), fatty acid-binding protein-1 (FABP-1, *p* = 0.04), and α-glutathione S-transferase (α-GST, *p* = 0.001) compared to the matched controls. The AIP cases with high PBG had increased FABP-1 levels in their plasma and urine compared to those with low PBG. In the AIP cases, KIM-1 correlated positively with PBG, CXCL10, CCL2, and TCC, and the liver marker α-GST correlated positively with IL-13, CCL2, and CCL4 (all *p* < 0.05). In conclusion, KIM-1, FABP-1, and α-GST could represent potential early indicators of renal and hepatic damage in AIP, demonstrating associations with porphyrin precursors and inflammatory markers.

## 1. Introduction

Acute intermittent porphyria (AIP) is an inherited disorder of heme synthesis in which decreased hydroxymethylbilane synthase (HMBS) enzyme activity leads to the accumulation of porphyrin precursors. Porphyria-associated kidney disease (PAKD) is seen in more than half of patients with symptomatic AIP [1,2,3], and more than half of patients with PAKD have hypertension [1]. CKD and other kidney diseases have been shown to be more common in patients with acute porphyria (of which AIP is the most common type) compared to reference populations and especially in AIP patients with elevated urine levels of the porphyrin precursor porphobilinogen (PBG) [4]. Although a relatively small proportion of symptomatic AIP patients (6.7%) [2] develop end-stage renal disease, this percentage is substantially higher than that observed in the general population [5]. Porphyrin precursors may induce oxidative stress and mitochondrial dysfunction in renal tubular cells, which is associated with tubular dysfunction in AIP [2].

Kidney mitochondria may be particularly vulnerable to oxidation by the porphyrin precursor delta-aminolevulinic acid (ALA), which may also be elevated in AIP [6]. Intracellularly, ALA goes through phosphate-catalyzed auto-enolization and becomes an oxidizing agent that reacts with iron and O_2_ and produces superoxide anion (O_2_^−^), hydroxyl radicals, and ALA radicals. ALA reduces iron in the proximity of oxygen and generates dioxovaleric acid (DOVA), a highly reactive oxidant. Growing evidence supports the hypothesis that ALA toxicity leads to damaged mitochondrial morphology, impaired protein expression, and the loss of transmembrane potential [6]. Also, ALA can cause vasoconstriction [1]. Examples of chronic tubulointerstitial nephropathy associated with nonspecific arteriosclerosis, chronic fibrous intima hyperplasia, and focal cortical atrophy have been seen histopathologically in PAKD [1,2].

Based on a study in which Pallet et al. incubated human renal epithelial cells with ALA and PBG, the authors proposed that porphyrin precursors promote renal epithelial cell apoptosis and produce phenotypic changes in proximal tubular cells, including epithelial-to-mesenchymal transition, endoplasmic reticulum stress, and kidney fibrosis [2]. The same study also observed increased levels of proinflammatory cytokines in the extracellular medium when the cells were incubated with ALA and PBG [2]. Collectively, these data suggest that porphyrin precursors can produce a proinflammatory and fibrogenic secretome [2]. In line with this observation, our previous case–control study demonstrated significantly elevated levels of cytokines in the plasma of 50 AIP patients compared with 50 matched controls [7] despite no significant differences in the number of other inflammatory diseases between the AIP cases and controls [7].

A genetic variant of the peptide transporter 2 (PEPT2) in the kidney tubuli is linked to the vulnerability to kidney damage in AIP, and this transporter affects how porphyrin precursors are reabsorbed into kidney tubule cells [8]. Previously, it was also suggested that in AIP, ischemic kidney injury may arise due to reduced blood flow caused by vasospasm, vasoactive substances, or hypovolemia [9,10].

While most heme synthesis takes place in the bone marrow (80%), heme synthesis in the liver (15%) is of particular importance for the disease process in AIP [6]. Moreover, heme synthesis also occurs in the kidneys, with it participating in the production of heme for certain detoxifying cytochromes and other heme-dependent proteins [6]. Heme synthesis in the kidneys is most active in the cortical proximal tubule, which is a metabolically active region that is particularly exposed to xenobiotics and endogenously produced compounds [6].

It is well known that AIP cases have a high risk of hepatocellular cancer (HCC) [11,12]. Interestingly, HCC in AIP cases is not associated with liver fibrosis [13]. Potential hypotheses for HCC development in AIP include a secondary somatic mutation, diminished antioxidant levels due to heme deficiency, ALA toxicity, and oxidative stress and inflammation [11]. Elevated urine PBG has been found to be associated with an increased risk for primary liver cancer in AIP [11]. It has been suggested that ALA, PBG, and/or porphyrins, along with tissue damage, may act as damage-associated molecular patterns (DAMPs) in AIP [7]. Both direct and indirect injury to hepatocytes may result in the release of cellular components into the bloodstream.

For the optimal prevention and treatment of AIP-related kidney and liver damage, early detection and understanding of the damage type are crucial. Traditional surrogate markers like serum creatinine and eGFR are insufficient for early kidney damage detection, and AST and ALT are late liver damage markers. More targeted diagnostic strategies using specific and sensitive modern kidney and liver damage markers, together with traditional markers, have the potential to improve patient outcomes. For example, kidney injury molecule-1 (KIM-1) outperforms serum creatinine as a predictor of kidney tubular histopathological changes in rat studies [14]. KIM-1 holds promise as an early biomarker of acute kidney injury [15]. It is a sensitive, specific marker for renal proximal tubular damage, and is associated with renal fibrosis and inflammation in chronic kidney disease [15]. Because of the challenges in predicting and preventing kidney and liver disease in AIP, our aim was to conduct an exploratory, descriptive, and hypothesis-generating analysis of novel kidney and liver biomarkers in samples that we had already obtained in a previously conducted case–control study. Because the samples in the case–control study were all collected at one time point for each patient, the current study is not able to determine whether these biomarkers can predict disease development in AIP, but instead, we aim to identify biomarkers of interest for evaluation in future studies. We expected to find distinctions in these markers between AIP cases and matched controls and among different AIP patient subgroups, as well as correlations with inflammatory markers and porphyrin precursors. In our case–control study of 50 AIP cases and 50 matched controls, we analyzed a range of new plasma (P) and urine (U) biomarkers that detect proximal, distal, and tubular kidney damage and hepatocellular damage, and we compared these with plasma cytokines, urine porphyrin precursors, traditional kidney and liver biomarkers, and dietary markers. Through this process, we identified potential early kidney and liver injury markers in AIP that may be important for patients.

## 2. Materials and Methods

### 2.1. Participants and Study Design

We conducted a case–control study of 50 genetically confirmed AIP cases and 50 controls randomly picked from the same population, matched for age, sex, and place of residence. The inclusion period was from September to November 2012. The participants lived in the Norwegian counties of Nordland, Troms, Trøndelag, and Oslo. As a practically possible approach for measurements of modern kidney and liver markers of the rare disease AIP, we included 50 AIP patients, of whom 35 were symptomatic (those who had ever had an AIP attack) and 15 were asymptomatic (those who had never had an AIP attack), and 50 matched controls. From the initial 50 AIP cases and 50 controls, there was an insufficient amount of urine samples available in the freezer for two of the AIP cases. As a result, these two AIP cases and their corresponding controls were omitted from the urine analysis for the modern kidney and liver biomarkers. We also divided these 50 AIP patients into the new consensus-based definition groups for AIP patients described in Stein et al. 2023 [16]. According to these new definitions, 2 had recurrent acute porphyria, 20 had sporadic acute porphyria, 3 were symptomatic high excreters, 7 had asymptomatic acute porphyria, 6 were asymptomatic high excreters, and 12 had latent porphyria but with the disclaimer that the self-reported previous AIP attacks were not always biochemically confirmed. High excretion of urine PBG/creatinine was defined by Stein et al. [16] as a urine PBG/creatinine ratio at least 4 times the upper limit of normal, which when applied to our study is urine PBG/creatinine > 6 µmol/mmol (i.e., 1.5 µmol/mmol × 4). We had previously stored EDTA plasma from all 50 cases and matched controls and stored urine from 48 cases and their matched controls at −80 °C. Each participant delivered blood and urine samples only once. None of the participants were experiencing an AIP attack when they had their samples drawn.

### 2.2. Ethical Considerations

All of the procedures in this trial were in accordance with the ethical standards of the institutional and national responsible committees on human research and the Helsinki Declaration. The Regional Committee for Medical and Health Research Ethics approved the study, approval number 2011/2197. The ClinicalTrials.gov identifier is NCT01617642. Written informed consent was obtained from all participants, and the study conforms to STROBE Guidelines. 

### 2.3. Liver and Kidney Biomarkers, PBG, ALA, and Other Biochemistry Tests

The kidney markers in urine were analyzed with the 9-plex Human Kidney Injury Magnetic bead panel 1 from Merck KGaA (Darmstadt, Germany) on a Luminex^®^ 200 system, and the results were given initially as ng/mL. This urine panel consisted of nine markers: collagen IV, tissue inhibitors of metalloproteinases-1 (TIMP-1), kidney injury molecule-1 (KIM-1), α-glutathione S-transferase (α-GST), fatty acid-binding protein (FABP-1), calbindin, chemokine-X-X-motif chemokine ligand 10 (CXCL10), trefoil factor-3 (TFF-3), and renin. All urine marker results were converted to picograms per milliliter (pg/mL) and corrected for urine creatinine (mmol) to compensate for differences in the concentration of urine, with the results expressed as pg/mmol creatinine.

The kidney markers in the plasma in pg/mL were analyzed on a Luminex^®^ 200 instrument system applying the 3-plex Human Kidney Injury Magnetic bead panel 4, Merck KGaA, catalogue number HKI4MAG-99K (plasma KIM-1, plasma Renin, and plasma FABP-1) and the liver marker Human Liver Injury Magnetic bead panel from Merck KGaA, catalogue number HLINJMAG-75-K (plasma α-GST). Though α-GST is a kidney damage marker in urine, it is a liver damage marker in plasma. The urine and plasma samples used for the new kidney markers had been stored frozen at −80 °C from 2012 and were analyzed in 2021 (urine) and 2022 (plasma).

Measurements in the urine of IgG and Alpha-1 microglobulin at Haukeland University Hospital in Bergen and Albumin at Nordland Hospital in Bodø were performed in 2012 with routine methods.

In 2012, the ADVIA^®^ 1800 system, provided by Siemens Medical Solutions Diagnostics in Japan, in combination with reagents supplied by Siemens Healthcare Diagnostics Limited, was employed for the quantification of various parameters, including serum (S) levels of bilirubin (µmol/L), gamma-glutamyl transferase (GGT, U/L), alkaline phosphatase (ALP, U/L), aspartate aminotransferase (AST, U/L), alanine aminotransferase (ALT, U/L), carbamide (mmol/L), creatinine (µmol/L), uric acid (µmol/L), and other routine biochemistry tests as previously outlined [7,17]. Plasma parathyroid hormone (PTH) levels, measured in picomoles per liter (pmol/L), were assessed using the ADVIA Centaur XP system (Siemens Healthcare Diagnostics, Tarrytown, NY, USA).

The analysis of HbA1c in EDTA blood was conducted using a Tosoh G8 high-performance liquid chromatography (HPLC) system and expressed in millimoles per mole (mmol/mol).

Additionally, assessments of plasma C-peptide and plasminogen activator inhibitor-1 (PAI-1), quantified in pg/mL, were conducted in 2012 through utilization of the Bio-Plex 200 system from Bio-Rad, along with a Bioplex Pro human diabetes immunoassay kit. Relevant quality control measurements were used during the assay procedures. Urine porphobilinogen (PBG) levels and urine aminolevulinic acid (ALA) were assessed at the laboratory at Nordland Hospital in Bodø, Norway, in 2012, using a kit from BioRad Laboratories (Munich, Germany) and reported in µmol/mmol creatinine. The measurement of total porphyrins was conducted as previously described and expressed as nmol/mmol creatinine [18].

### 2.4. Multiplex Immunoassays

Cytokines in EDTA plasma were examined using multiplex technology in a case–control study involving 50 AIP cases, as detailed in a prior investigation [7]. Additionally, assessments of plasma C-peptide and plasminogen activator inhibitor-1, reported as pg/mL, were conducted using the Bio-Plex 200 system from Bio-Rad and a Bioplex Pro human diabetes immunoassay kit. Quality control measures were implemented during the assay procedures.

### 2.5. Enzyme Immunoassays

Complement activation biomarker soluble C5b-9 terminal complement complex (TCC) was determined in EDTA plasma using ELISA, following previously established protocols, and quantified as complement arbitrary units per milliliter (CAU/mL) [19]. Optical density measurements were acquired through utilization of an MRX microplate reader (Dynex Technologies, Denkendorf, Germany) [20].

### 2.6. Indexes, Scores, and Ratios

The absolute glomerular filtration rate was calculated for each study participant using the Chronic Kidney Disease Epidemiology Collaboration (CKD-EPI) creatinine equation (mL/min) and the cystatin equation (mL/min), adjusting for body surface area by including the measured height and weight of each participant in the equation [21]. In addition, we calculated the estimated GFR (eGFR) using the creatinine equation (mL/min/1.73 m^2^), as it is used for categorizing the risk of CKD as defined by kdigo.org 2012 [22,23]. The AST to Platelet Ratio Index (APRI) [24], the Liver Fibrosis Index-4 (FIB-4) [25], and the Enhanced Liver Fibrosis (ELF) [26] scores were calculated as previously described. APRI = (AST)/upper limit of normal of (AST)) × 100/platelet count as 10^9^/L) [24]. FIB-4 = (age × AST)/(platelets × (sqr.(ALT)), with age in years, AST and ALT in U/L, and platelet count in 10^9^/L. ELF score = 2.278 + 0.851 ln (cHA) + 0.751 ln (cPIINP) + 0.394 ln (cTIMP-1), where HA= hyaluronic acid, PIINP = amino terminal propeptide of type III procollagen, TIMP-1 = tissue inhibitor of metalloproteinases, and c = concentration [26]. HA, PIINP, and TIMP-1 for the ELF test were analyzed on the Advia Centaur immune assay system in 2015.

### 2.7. Recording of Diet, Smoking, and Anthropometric Measurements

For both the AIP cases and controls, a comprehensive inquiry was conducted to gather information on symptoms, dietary habits, and smoking history. Pack–years were defined as the number of packs of 20 cigarettes smoked per day multiplied by the total years as a smoker. Additionally, a 7-day diet logbook was utilized to collect data on the consumption of grams per day of dietary items, including vegetables, carbohydrates, proteins, fat, and different nutrients. These diet logbooks were scanned using the Teleform program version 6.0 by Datascan, Oslo, Norway. Daily dietary intake calculations were performed using the food database and software system (KBS) developed by the Department of Nutrition at the University of Oslo. Of the 50 AIP cases, 47 of them filled out the diet logbook. The presented dietary data are, therefore, from 47 AIP cases. Body weight, length, and waist-to-hip ratio were assessed by trained nurses following WHO guidelines.

### 2.8. Statistical Analysis

The Wilcoxon signed-rank test for matched pairs was used for most of the data, comparing AIP cases versus matched controls. Fisher’s exact test was used for categorical variables. The Mann–Whitney U-test was used when comparing a group of asymptomatic AIP cases versus a group of symptomatic AIP cases or AIP cases with high versus low PBG or ALA levels. Spearman’s rank correlation coefficient was used for the AIP cases to calculate correlation coefficients (ρ) and two-tailed *p*-values. In the correlation matrixes, the color coding is deep blue for ρ-values approaching 1 and bright red for ρ-values of −1, while ρ values of 0.00 are white. Statistical significance was defined as *p* < 0.05. For plasma FABP-1, 17 of 100 samples were out of range and below what the test could detect or out of range (<OOR). As the lower limit of detection was 34 and 35, the values for these seventeen cases were randomly assigned a number for FABP-1 between 0–34 or 0–35. Even when using the same assay kit and equipment, and the same sample handling, as was the case in our study, minor differences in the lower limit of detection (LLD) can be seen for reasons such as minor analytical variability and random chance since the analyses of samples regarding the modern liver and kidney markers on 50 AIP cases and 50 matched controls were performed in three different runs within a few days. For urine calbindin, 22 of 96 were out of range and below what the test could detect (<OOR), and values in these 22 cases were therefore randomly assigned a number for calbindin between 0 and the lower limit of detection (LLD 0.1–0.6). For urine CXCL10, 15 of 96 samples were out of range and below what the test could detect (<OOR), and these 15 cases were therefore randomly assigned a number for CXCL10 between 0–LLD = 0.01. For urine renin, 39 of 96 were <OOR, but for 20 of them, the LLD was 0, resulting in a reported value of 0. For the other 19, the limit of detection was 0.02; hence, these 19 persons were randomly assigned an LLD value between 0 to 0.02. For urine alpha-1-macroglobulin, 31 of the 50 AIP cases and 37 of the 50 matched controls had values < 0.5 and were given a randomly drawn number below 0.5 for the figures and statistics. For urine IgG, 36 of the 50 AIP cases and 35 of the 50 matched controls had values < 0.7 and were given a randomly drawn number below 0.7 for the figures and statistics. The results were analyzed using GraphPad Prizm versions 9 and 10 from GraphPad Software Inc. (San Diego, CA, USA). The cluster/heatmap figure (Figure A8) was generated using Python (Python 3.10.12 (main, 11 Jun 2023, 05:26:28) [GCC 11.4.0] on Linux) and was further edited in Adobe Illustrator version 28 (64 bit), Adobe Inc. (San Jose, CA, USA). The heatmap was generated using single linkage and cosine distance metrics. The expression values are scaled as Z-scores and displayed using a color scale. The graphical abstract was made with BioRender.com.

## 3. Results

### 3.1. Baseline Characteristics of AIP Cases and Matched Controls

The baseline characteristics of the 50 AIP cases and 50 controls are shown in Table 1. Of the 50 AIP cases, 35 were symptomatic and 15 were asymptomatic. Of the 48 AIP cases with urine samples for measuring novel kidney markers, 33 were symptomatic, and 15 were asymptomatic. Kidney function was similar in the AIP cases compared with the controls when absolute GFR was determined by the conventional kidney markers cystatin C and serum creatinine (Table 1). Mean arterial pressure was similar in the AIP cases and the controls (Table 1). Furthermore, body mass index was similar in both groups, as previously shown for this study population [27].

### 3.2. Kidney and Liver Markers in Plasma and Urine in AIP Cases Compared with Controls

**Figure 1:**
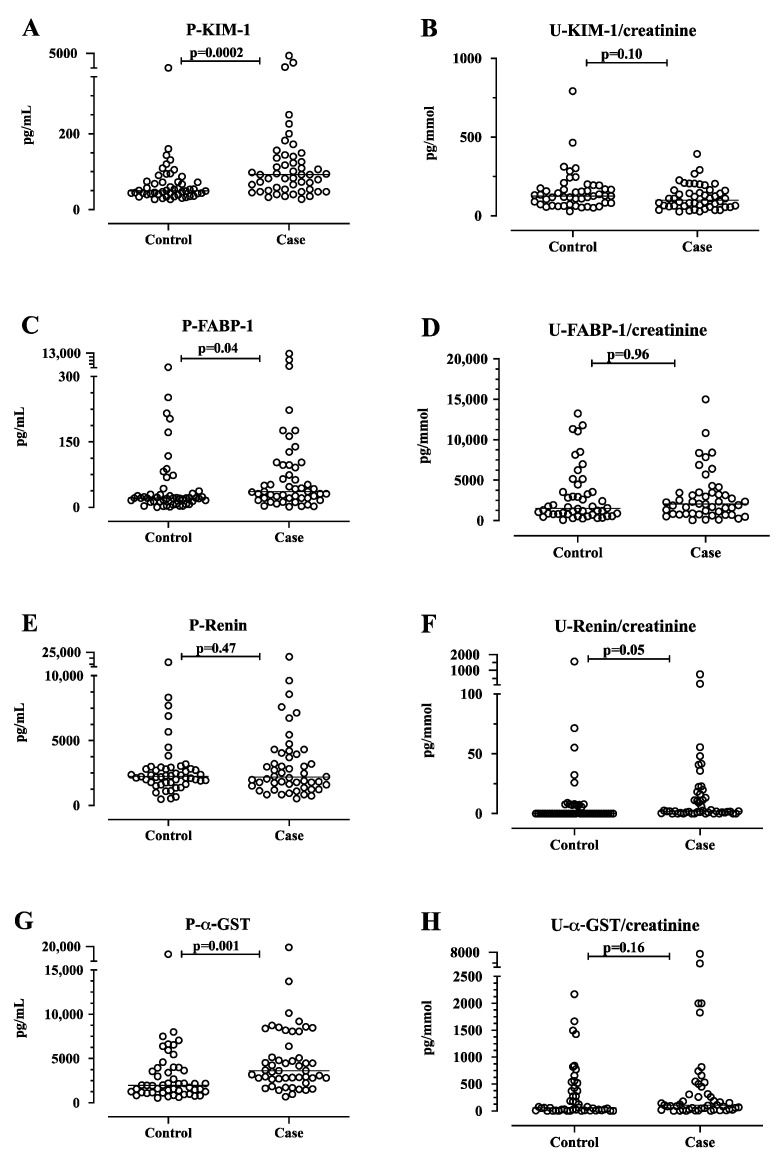
The plasma and urine levels of kidney injury molecule-1 (KIM-1), fatty acid-binding protein-1 (FABP-1), renin, and glutathione S-transferase alpha (α-GST) in acute intermittent porphyria (AIP) cases compared with their matched controls (*n* = 48 in urine and *n* = 50 in plasma). The KIM-1 (**A**), FABP-1 (**C**), renin (**E**), and α-GST (**G**) in plasma are expressed as pg/mL. The KIM-1 (**B**), FABP-1 (**D**), renin (**F**), and α-GST (H) in urine are expressed as pg/mmol creatinine. The results are shown as scatter plots with a horizontal line for the median. The data were analyzed through use of the Wilcoxon signed-rank test for matched-pairs. The *p*-values are exact, two-tailed values. P, plasma; U, urine.

**Figure A1:**
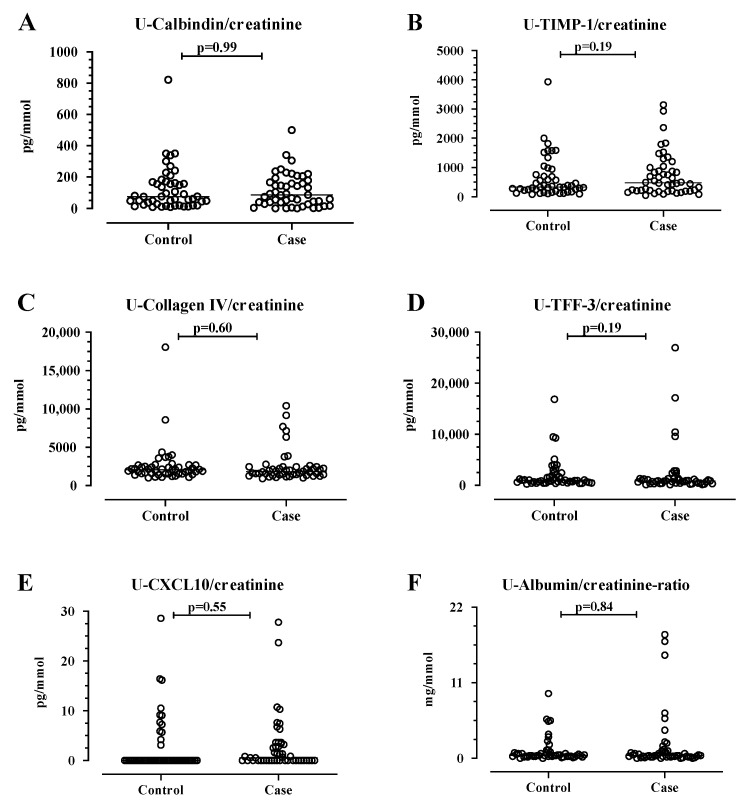
The urine levels of calbindin, tissue inhibitor of metalloproteinases (TIMP-1), collagen IV, trefoil factor-3 (TFF-3), chemokine (C-X-C), motif ligand 10 (CXCL10), and albumin/creatinine ratio in the acute intermittent porphyria (AIP) cases (*n* = 48) compared with their matched controls (*n* = 48). The Calbindin/creatinine (**A**), TIMP/creatinine (**B**), Collagen IV/creatinine (**C**), TFF-3/creatinine (**D**), and CXCL10/creatinine (**E**) in urine are expressed as pg/mmol creatinine. The Albumin/creatinine-ratio (**F**) is expressed as mg/mmol creatinine. The results are shown as scatter plots with a horizontal line for the median. The data were analyzed using the Wilcoxon signed-rank test for matched pairs. The *p*-values are exact, two-tailed values. U, urine.

The median plasma level of the tubular damage marker KIM-1 was significantly higher (median 92 pg/mL, IQR 52–137) in the 50 AIP cases compared to the matched controls (median 50 pg/mL, IQR 40–73, *p* = 0.0002, Figure 1). The mean age for the AIP cases with the highest quartile of plasma KIM-1 (75 percentile > 137 pg/mL) was 60.6 years compared to 50.6 years for all 50 AIP cases. The mean age for the controls with the highest quartile of P-KIM-1 (75 percentile > 73.3 pg/mL) was 57.3 years. The mean level of urine PBG was 2.5-fold higher in the 12 AIP cases in the highest quartile for plasma KIM-1 compared to the mean of all cases. Of these 12 AIP cases of the highest KIM-1, 2 had known CKD compared to one of the controls. Similarly, the plasma level of proximal tubular damage marker FABP-1 was significantly higher in the AIP cases (median 36 pg/mL, IQR 21-97) compared to the matched controls (median 21 pg/mL, IQR 13-33, *p* = 0.04, Figure 1). We found no significant difference (*p* > 0.05) in the plasma renin levels between the AIP cases and the matched controls (Figure 1). Furthermore, we did not find any significant differences in the following urine markers between the AIP cases and the matched controls: KIM-1, FABP-1, α-GST (Figure 1), calbindin, TIMP-1, collagen IV, TFF-3, or CXCL10 (Figure A1). There was a non-significant difference in urine renin between the AIP cases (median of 2.0, IQR 1.0–18.2) and the matched controls (median of 0.0, IQR 0-6.4, *p* = 0.05, Figure 1).

The plasma liver marker α-GST was significantly higher in the AIP cases (median 3626 pg/mL, IQR 2706-6799) compared with the matched controls (median 1982 pg/mL, IQR 1163–3954, *p* = 0.0012, Figure 1).

**Figure 2:**
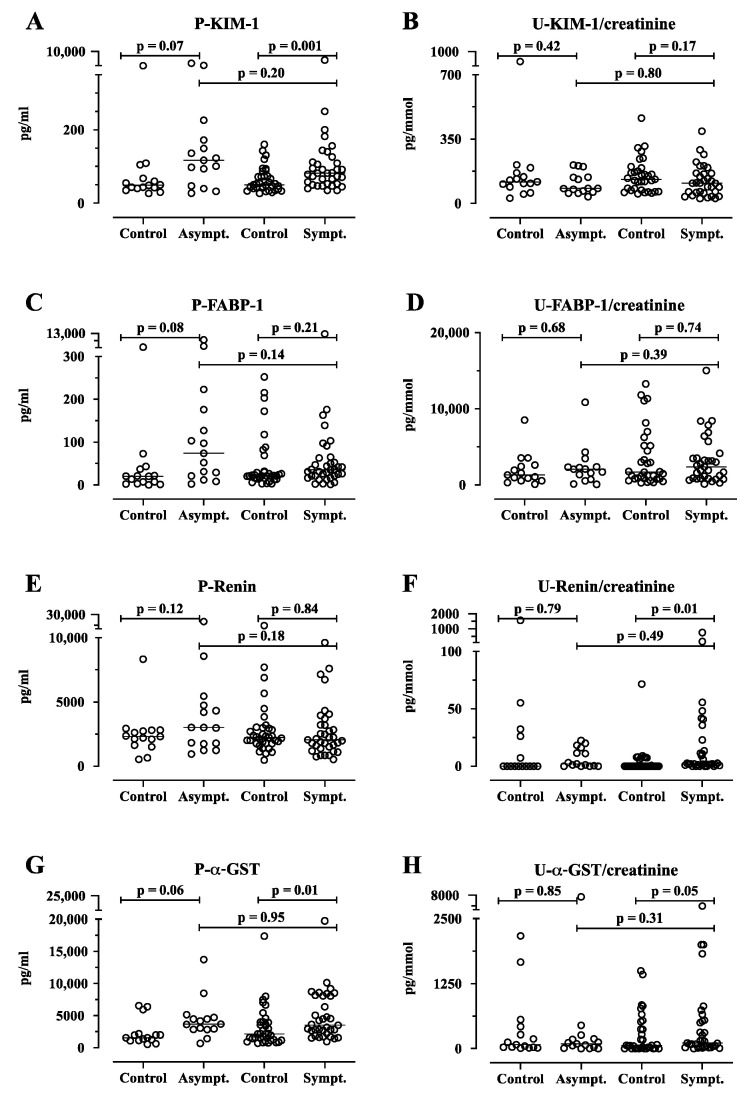
The plasma and urine levels of kidney injury molecule (KIM-1), fatty acid-binding protein (FABP-1), renin, and alpha-glutathione S-transferase (α-GST) in 15 asymptomatic (Asympt.) and 35 symptomatic (Sympt.) acute intermittent porphyria (AIP) cases and matched controls (*n* = 50 for plasma and 48 for urine). The KIM-1 (**A**), FABP-1 (**C**), renin (**E**), and α-GST (**G**) in plasma are expressed as pg/mL. The KIM-1 (**B**), FABP-1 (**D**), renin (**F**), and α-GST (**H**) in urine are expressed as pg/mmol creatinine. The results are shown as scatter plots with medians. The paired case–control data were analyzed using the Wilcoxon signed-rank test for matched pairs. The asymptomatic and symptomatic AIP cases were compared using the Mann–Whitney U-test. The *p*-values are exact, two-tailed values. P, plasma; U, urine.

**Figure 3:**
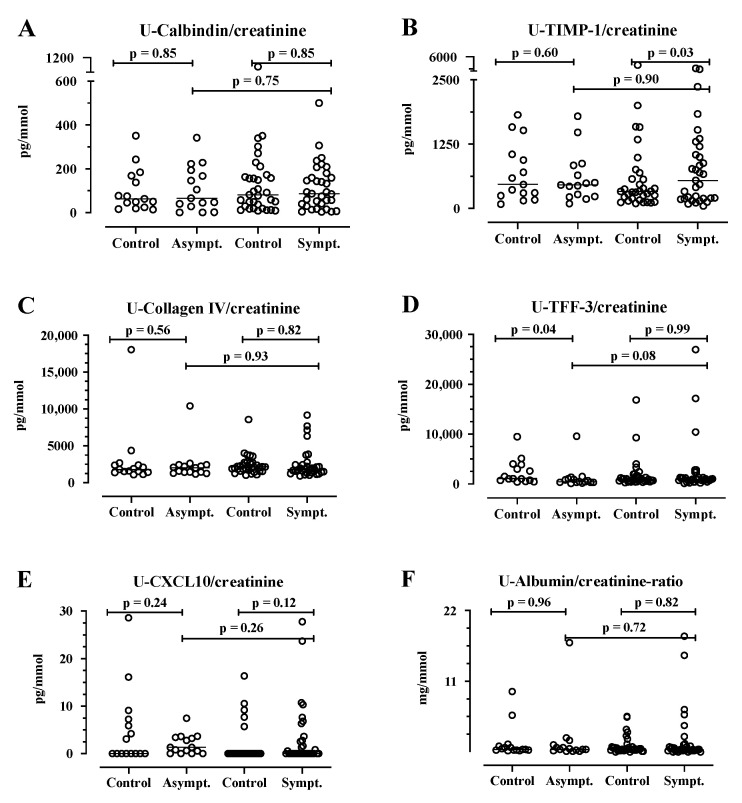
The urine levels of calbindin, tissue inhibitor of metalloproteinases (TIMP-1), collagen IV, trefoil factor-3 (TFF-3), chemokine (C-X-C), motif ligand 10 (CXCL10), and albumin in 15 asymptomatic (Asympt.) and 33 symptomatic (Sympt.) acute intermittent porphyria (AIP) cases and matched controls (*n* = 48 pairs). The Calbindin/creatinine (**A**), TIMP/creatinine (**B**), Collagen IV/creatinine (**C**), TFF-3/creatinine (**D**), and CXCL10/creatinine (**E**) in urine are expressed as pg/mmol creatinine. The Albumin/creatinine-ratio (**F**) is expressed as mg/mmol creatinine. The results are shown as scatter plots with a horizontal line for the median. The paired case–control data were analyzed using the Wilcoxon signed-rank test for matched pairs. The asymptomatic and symptomatic AIP cases were compared using the Mann–Whitney U-test. The *p*-values are exact, two-tailed values. U, urine.

The kidney damage marker KIM-1 in plasma was significantly higher in the symptomatic AIP cases (*n* = 35, median 82 pg/mL, IQR 53–112) compared with their matched controls (*n* = 35, median 50 pg/mL, IQR 40–74, *p* = 0.0013, Figure 2). Furthermore, the plasma levels of FABP-1 and renin did not differ significantly between the symptomatic AIP cases and the matched controls (*p* >0.05, Figure 2). Urine renin levels were significantly higher in symptomatic AIP cases (*n* = 33, median 2 pg/mL, IQR 1.0–18) compared with their matched controls (*n* = 33, median 0.0 pg/mL, IQR 0.0–6.4, *p* = 0.01, Figure 2). Furthermore, urine TIMP-1 was significantly higher in the symptomatic AIP cases (median 546 pg/mL, IQR 191–1123), compared to their matched controls (median 330 pg/m, IQR 180–634, *p* = 0.03, Figure 3). Additionally, the urine levels of KIM-1, FABP-1 (Figure 3), calbindin, collagen IV, TFF, and CXCL10 (Figure A1) did not differ significantly between the symptomatic AIP cases and the matched controls (*p* > 0.05). For α-GST in urine, there was a non-significant difference between the symptomatic AIP (*n* = 33) cases (median 110, IQR 27–537) and the matched controls (median of 59, IQR 3-446, *p* = 0.05, Figure 2).

The liver marker α-GST in plasma was higher in the symptomatic AIP cases (median 3505 pg/mL, IQR 2227–8073) compared with their matched controls (median 2160 pg/mL, IQR 1167–3983, *p* = 0.01, Figure 2). Although it did not reach statistical significance, this pattern was similar to the relationship between the asymptomatic AIP cases (*n* = 15, median 3678 pg/mL, IQR 2850–4717) and their matched controls (*n* = 15, median 1553 pg/mL, IQR 1151–2178, *p* = 0.06, Figure 2).

**Figure 4:**
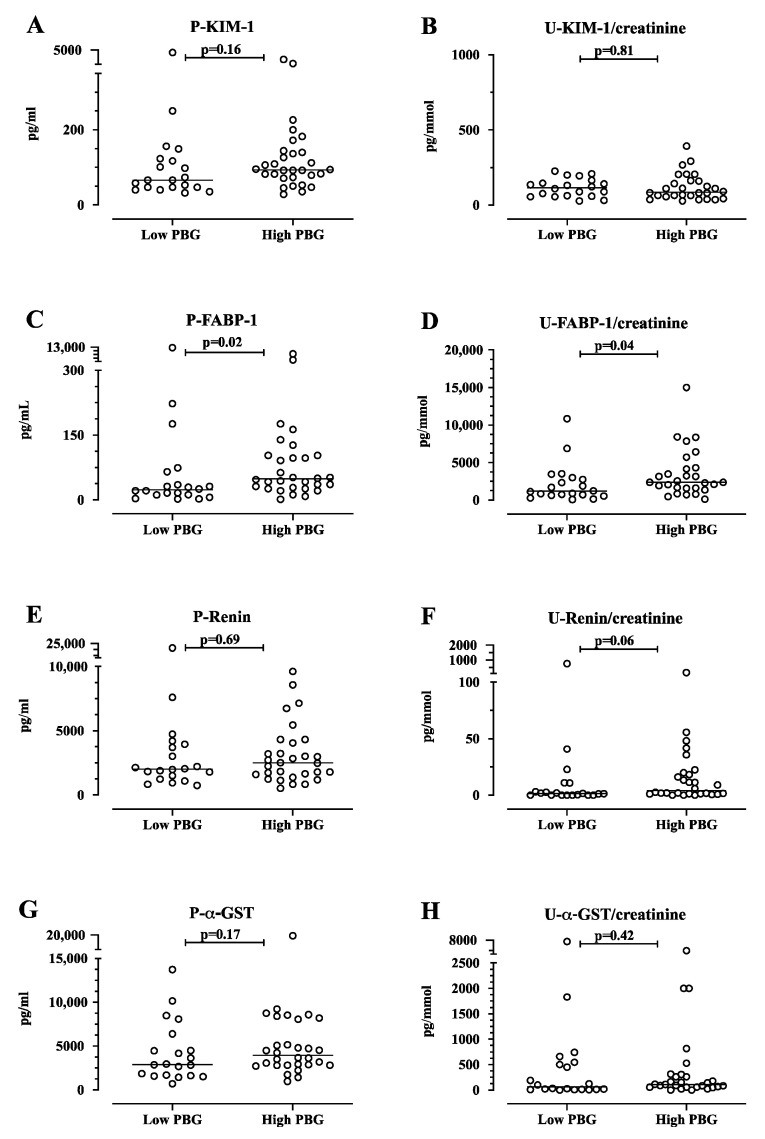
The plasma and urine levels of kidney injury molecule-1 (KIM-1), fatty acid-binding protein (FABP-1), renin, and alpha-glutathione S-transferase (α-GST) in acute intermittent porphyria (AIP) cases with low urine porphobilinogen (PBG) compared with the AIP cases with high PBG. A low PBG level was defined as a value ≤1.5 μmol PBG/mmol creatinine, the reference limit for this assay. A high urine PBG/mmol creatinine level was defined as a value > 1.5 μmol PBG/mmol creatinine, the normal upper reference level. KIM-1 (**A**), FABP-1 (**C**), renin (**E**), and α-GST (**G**) in plasma are expressed as pg/mL. KIM-1 (**B**), FABP-1 (**D**), renin (**F**), and α-GST (H) in urine are expressed as pg/mmol creatinine. The results are shown as scatter plots with a horizontal line for the median. The data were analyzed using the Mann–Whitney U-test. The *p*-values are exact, two-tailed values. P, plasma; U, urine.

The plasma levels of KIM-1, FABP-1, and renin did not differ significantly between the symptomatic and asymptomatic AIP cases (*p* > 0.05, Figure 2). Furthermore, we did not find any significant differences in the following urine markers between the symptomatic AIP cases compared with the asymptomatic AIP cases: KIM-1 FABP-1, renin, α-GST (Figure 2), calbindin, TIMP-1, collagen IV, TFF, or CXCL10 (Figure 4).

Plasma α-GST did not differ significantly between the symptomatic and asymptomatic AIP cases (*p* > 0.05, Figure 2).

### 3.3. Kidney and Liver Markers in Plasma and Urine in AIP Cases with High Versus Low Urine PBG/Creatinine Levels and Correlations with Porphyrins and Porphyrin Precursors

**Figure A2:**
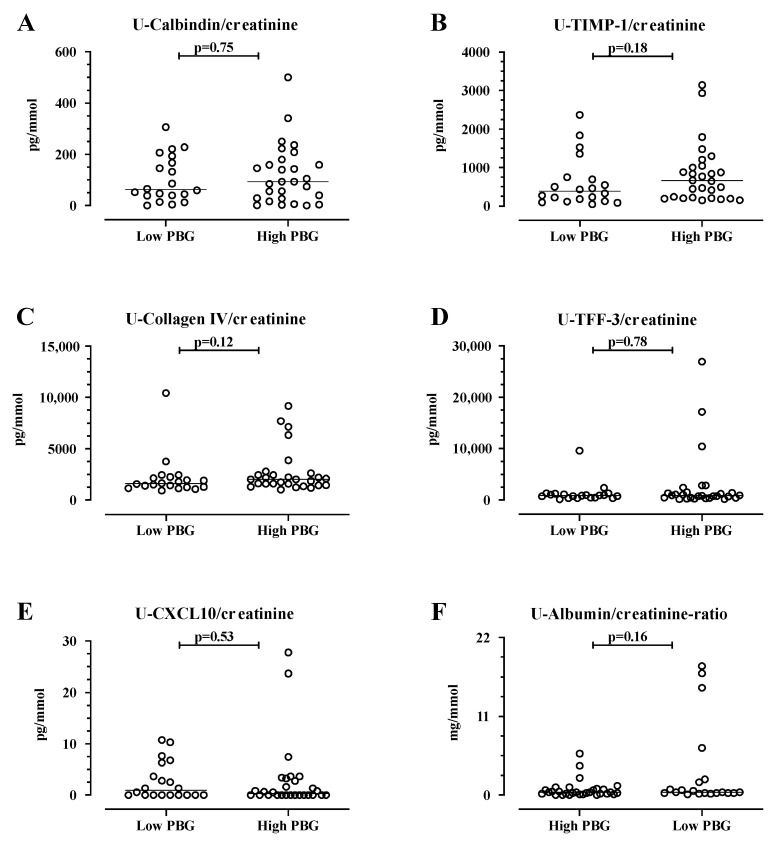
The urine levels of calbindin, tissue inhibitor of metalloproteinases (TIMP-1), collagen IV, trefoil factor-3 (TFF-3), chemokine (C-X-C) motif ligand 10 (CXCL10), and albumin in acute intermittent porphyria (AIP) cases with low urine porphobilinogen (PBG) compared with AIP cases with high urine PBG levels. A low urine PBG level was defined as a value ≤1.5 μmol PBG/mmol creatinine, the reference limit for this assay. A high urine PBG/mmol creatinine level was defined as a value > 1.5 μmol PBG/mmol creatinine, the normal upper reference level. The Calbindin/creatinine (**A**), TIMP/creatinine (**B**), Collagen IV/creatinine (**C**), TFF-3/creatinine (**D**), and CXCL10/creatinine (**E**) in urine are expressed as pg/mmol creatinine. The Albumin/creatinine-ratio (**F**) is expressed as mg/mmol creatinine. The data were analyzed using the Mann–Whitney U-test. The *p*-values are exact, two-tailed values. The results are shown as scatter plots with a horizontal line for the median. U, urine.

The kidney damage marker FABP-1 in plasma was higher in the group of AIP cases with high urine PBG (*n* = 30, median 49 pg/mL, IQR = 28–103) compared with the group of AIP cases with low urine PBG (*n* = 20, median 23 pg/mL, IQR 12–58, *p* = 0.02, Figure 4). Similarly, FABP-1 in the urine was higher in the group of AIP cases with high urine PBG (*n* = 28, median 2364 pg/mmol, IQR 1574–4263) compared with the group of AIP cases with low urine PBG (*n* = 20, median 1177 pg/mL, IQR 654–2926, *p* = 0.04, Figure 4). Plasma levels of KIM-1 and renin and urine levels of KIM-1, renin, and α-GST did not differ significantly between the AIP cases with high versus low urine PBG (Figure 4). The liver biomarker plasma α-GST did not differ significantly between the AIP cases with high versus low urine PBG (Figure 4). Furthermore, urine calbindin, TIMP-1, collagen IV, TFF, CXCL10, and albumin/creatinine-ratio did not differ significantly between the AIP cases with high versus low urine PBG (Figure A2).

**Figure 5:**
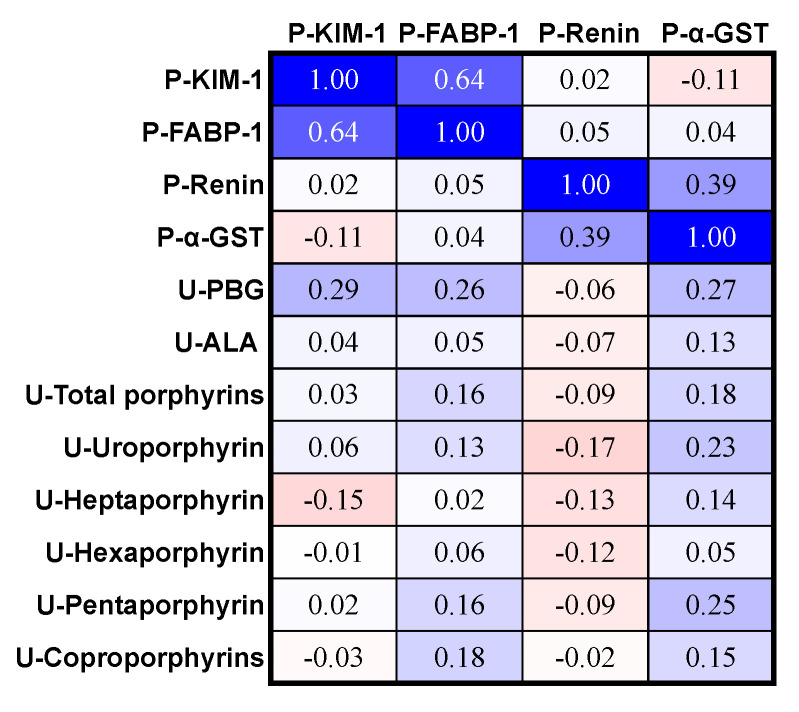
Correlation matrix of urine porphyrin precursors, urine porphyrins, and novel plasma biomarkers of kidney and liver damage in the acute intermittent porphyria group (*n* = 50). The urine biomarkers are expressed as the ratio to mmol creatinine in urine. The figure shows the Spearman ρ-values. ρ ≤ −0.28 and ρ ≥ 0.28 correspond to statistically significant *p*-values < 0.05. The Q values are colored on a scale from deep blue if +1 to bright red if −1, with white being zero. U, urine; P, plasma; KIM-1, kidney injury molecule-1; FABP-1, fatty acid-binding protein-1; α-GST, alpha-glutathione S-transferase.

The urinary PBG levels correlated significantly and positively with KIM-1 (Figure 5). Furthermore, the correlation between urinary PBG and FABP-1 in the plasma was not significant (Spearman ρ = 0.26, *p* = 0.06, Figure 5). Renin levels in the plasma were not significantly correlated with urinary PBG levels (Figure 5). The level of urine ALA did not correlate significantly with the plasma levels of KIM-1, FABP-1, or renin (Figure 5). The urinary levels of total porphyrins, uroporphyrins, heptaporphyrin, hexaporphyrin, pentaporphyrin, and coproporphyrin were not significantly correlated with the plasma levels of KIM-1, FABP-1, or renin (Figure 5).

The correlation between urine PBG versus plasma α-GST did not reach significance (*p*-value = 0.06, positive ρ, Figure 5). The urine levels of ALA, total porphyrins, uroporphyrin, heptaporphyrin, hexaporphyrin, pentaporphyrin, and coproporphyrin were not significantly correlated with plasma α-GST (Figure 5).

**Figure 6:**
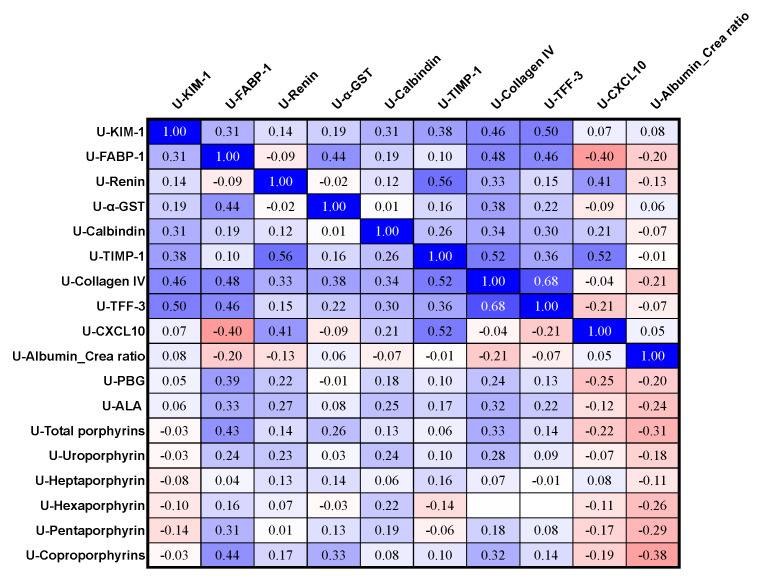
Correlation matrix of urine porphyrin precursors, urine porphyrins, and novel urine kidney-related biomarkers in the AIP cases (*n* = 48). The figure shows ρ-values, and ρ-values ≤ −0.28 and ρ ≥ 0.28 correspond to statistically significant *p*-values < 0.05. The ρ-values are colored on a scale from deep blue if +1 to bright red if −1, with white being zero. All urine levels were corrected for urine creatinine. KIM-1, kidney injury molecule-1; FABP-1, fatty acid-binding protein-1; α-GST, glutathione S transferase alpha; U, urine; P, plasma.

Urine PBG levels correlated significantly and positively with urine FABP-1 (*p* = 0.007, Figure 6), but urine PBG did not correlate significantly with urine levels of KIM-1, renin, α-GST, Calbindin, TIMP-1, collagen IV, TFF-3, CXCL10, and the albumin/creatinine ratio. The urine ALA levels correlated significantly and positively with urine FABP-1 (*p* = 0.02) and urine collagen IV (*p* = 0.03, Figure 6) but did not correlate significantly with urine KIM-1, renin, α-GST, Calbindin, TIMP-1, TFF-3, CXCL10, and albumin/creatinine ratio. Urine total porphyrins correlated significantly and positively with urine FABP-1 (*p* = 0.002) and urine Collagen IV (*p* = 0.02). Furthermore, urine total porphyrins correlated significantly and negatively with the urine albumin/creatinine ratio (*p* = 0.03). The total porphyrins did not correlate significantly with the urinary levels of KIM-1, renin, α-GST, calbindin, TIMP-1, TFF, and CXCL10. The urinary levels of uroporphyrin, heptaporphyrin, and hexaporphyrin levels did not correlate significantly with any of the following urine markers: KIM-1, FABP-1, renin, α-GST, Calbindin, TIMP-1, collagen IV, TFF-3, CXCL10, and albumin/creatinine ratio. Urine pentapophyrin levels correlated significantly and positively with urine FABP-1 (*p =* 0.03, Figure 6) and negatively with the urine albumin/creatinine ratio (*p* = 0.04). However, urine pentaporphyrin did not correlate significantly with urine KIM-1, renin, α-GST, Calbindin, TIMP-1, collagen IV, TFF-3, and CXCL10. Urine coproporphyrins correlated significantly and positively with urine FABP-1 (*p* = 0.002), urine α-GST (*p* = 0.02), and urine collagen IV (*p* = 0.02) (Figure 6) and negatively with the urine albumin/creatinine ratio (*p* = 0.008). However, urine coproporhyrins did not correlate significantly with urinary levels of KIM-1, renin, Calbindin, TIMP-1, TFF-3, and CXCL10.

### 3.4. Correlations between Plasma Cytokines and a Plasma Liver Function Marker in AIP

**Figure 7:**
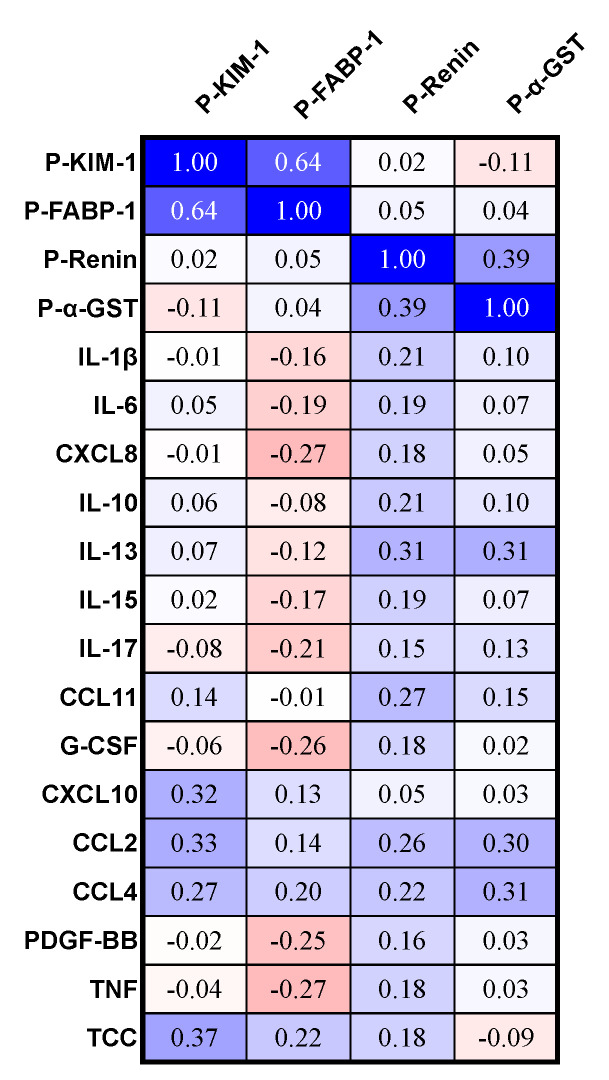
Correlation matrix of plasma cytokines, the terminal complement complex (TCC), plasma kidney-related biomarkers, and a plasma liver-related marker in AIP the cases (*n* = 50, AIP cases). The figure shows ρ-values, and ρ-values ≤ −0.28 and ρ ≥ 0.28 correspond to a statistically significant *p*-value < 0.05. The ρ-values are colored on a scale from deep blue if +1 to bright red if −1, with white being zero. KIM-1, kidney injury molecule-1; FABP-1, fatty acid-binding protein-1; α-GST, alpha-glutathione S-transferase; P, plasma.

Plasma IL-13, CCL2, and CCL4 correlated significantly and positively with plasma α-GST (*p* = 0.03 for all, Figure 7) but not with the other eleven cytokines or TCC (Figure 7).

### 3.5. Correlations between Plasma Cytokines and Plasma Kidney Markers in AIP

AIP cases with serum cystatin C above the reference levels had significantly higher levels of plasma cytokines CCL2 and CXCL10 compared to the AIP cases with normal serum Cystatin C levels (Table 2). The plasma cytokines CXCL10 and CCL2 correlated significantly and positively with plasma KIM-1 (*p* = 0.03, *p* = 0.02, Figure 7). However, the plasma cytokines IL-1ß, IL-6, CXCL8, IL-10, IL-13, IL-15, IL-17, CCL11, G-CSF, CCL4, PDGF-BB, and TNF did not correlate significantly with plasma KIM-1 (Figure 7). The terminal complement complex (TCC) correlated significantly and positively with plasma KIM-1 (*p* = 0.009, Figure 7). FABP-1 in plasma did not correlate significantly with any of the shown cytokines or with TCC (Figure 7). Plasma renin correlated significantly and positively with IL-13 (*p* = 0.03, Figure 7), but it did not correlate with the other thirteen cytokines or TCC (Figure 7).

### 3.6. Correlations between Plasma KIM-1 and Age and between Absolute GFR and Age

**Figure 8:**
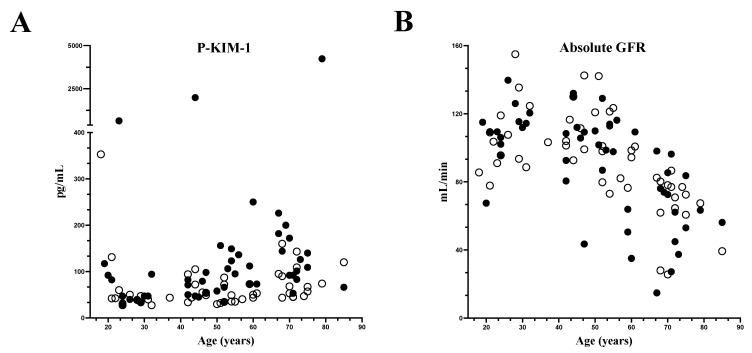
Correlation between plasma kidney injury molecule-1 (P-KIM-1) and age in years (**A**) and absolute GFR and age in years (**B**) in the AIP cases (filled black dots) and the matched controls (open circles). The absolute GFR is based on the CKD-EPI cystatin C equation. Each figure shows one sample per patient. Among the 50 AIP cases, there are 35 symptomatic and 15 asymptomatic cases.

In the AIP cases, plasma KIM-1 correlated significantly and positively with age in years, (Spearman ρ = 0.46, *p* = 0.0008, Figure 8A, filled black dots). In the matched controls, plasma KIM-1 correlated significantly and positively with age in years, (Spearman ρ = 0.33, *p* = 0.02, Figure 8A, open circles). In the AIP cases, absolute GFR based on the cystatin C equation correlated significantly and negatively with age in years (ρ = −0.62, *p* < 0.0001, Figure 8B, filled black dots). In the matched controls, absolute GFR based on the cystatin C equation correlated significantly and negatively with age in years (ρ = −0.63, *p* = <0.0001, Figure 8B, open circles). Furthermore, we divided the AIP cases into those with low GFR < 60 mL/min and those with GFR ≥ 60 mL/min, calculated as absolute GFR using the cystatin C equation. AIP cases with a GFR < 60 (*n* = 9) had a median KIM-1 of 113.5 pg/mL and median age of 69 years, while the AIP cases with GFR ≥ 60 mL/min (*n* = 41) had a median KIM-1 of 82.5 pg/mL and a median age of 48.5 years.

### 3.7. Correlations between the Novel Kidney Marker Plasma KIM-1 and the Conventional Serum Kidney Markers Absolute GFR Using Cystatin C and Creatinine Measurements

**Figure A3:**
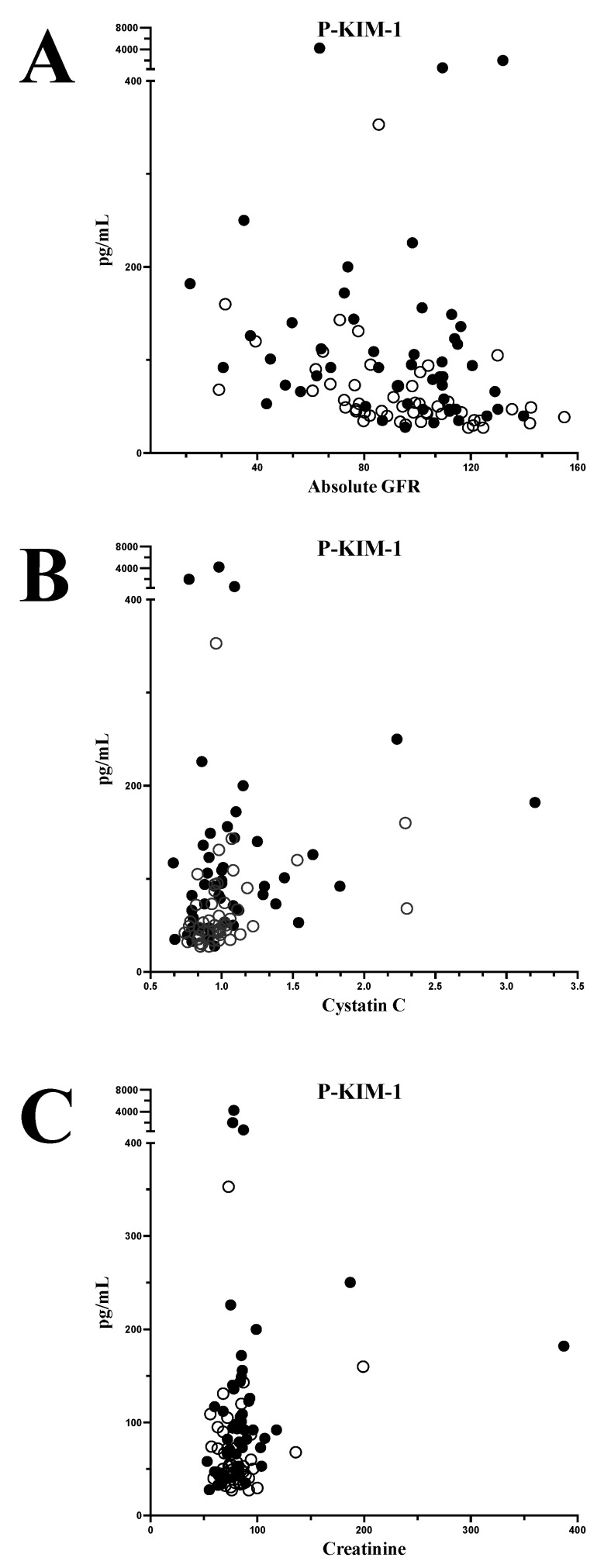
Correlations between plasma (P) KIM-1 versus conventional kidney markers. (**A**) Correlation between plasma kidney injury molecule-1 (P-KIM-1) and absolute CKD-Epi GFR (mL/min) based on the cystatin C equation for the AIP cases (ρ = −0.28, *p* = 0.045) and the controls (ρ = −0.54, *p* < 0.0001), (**B**) plasma KIM-1 and serum cystatin C (mg/L) for the AIP cases (ρ = 0.37, *p* = 0.008) and the controls (ρ = 0.35, *p* = 0.01), and (**C**) plasma KIM-1 and serum creatinine (µmol/L) for the AIP cases (ρ = 0.40, *p* = 0.004) and the controls (ρ = −0.11, *p* = 0.44), and in the AIP cases (filled black dots) and in the matched controls (open circles).

In the AIP cases, plasma KIM-1 correlated significantly and negatively with the absolute CKD-EPI GFR based on the cystatin C value (ρ = −0.28, *p* = 0.045, Figure A3, Appendix A). In the matched controls, plasma KIM-1 correlated significantly and negatively with the absolute GFR (ρ = −0.54, *p* < 0.0001, Figure A3, Appendix A). In the AIP cases, plasma KIM-1 correlated significantly and positively with the serum cystatin C value (ρ = 0.37, *p* = 0.008, Figure A3, Appendix A), and in the matched controls, plasma KIM-1 correlated significantly and positively with cystatin C (ρ = 0.35, *p* = 0.01, Figure A3, Appendix A). In the AIP cases, plasma KIM-1 correlated significantly and positively with serum creatinine (ρ = 0.40, *p* = 0.004, Figure A3, Appendix A), and in the matched controls, plasma KIM-1 did not correlate significantly with serum creatinine (ρ = −0.11, *p* = 0.44, Figure A3, Appendix A).

### 3.8. Urine Alpha-1 Microglobulin and Urine IgG in AIP Cases and Matched Controls

**Figure A4:**
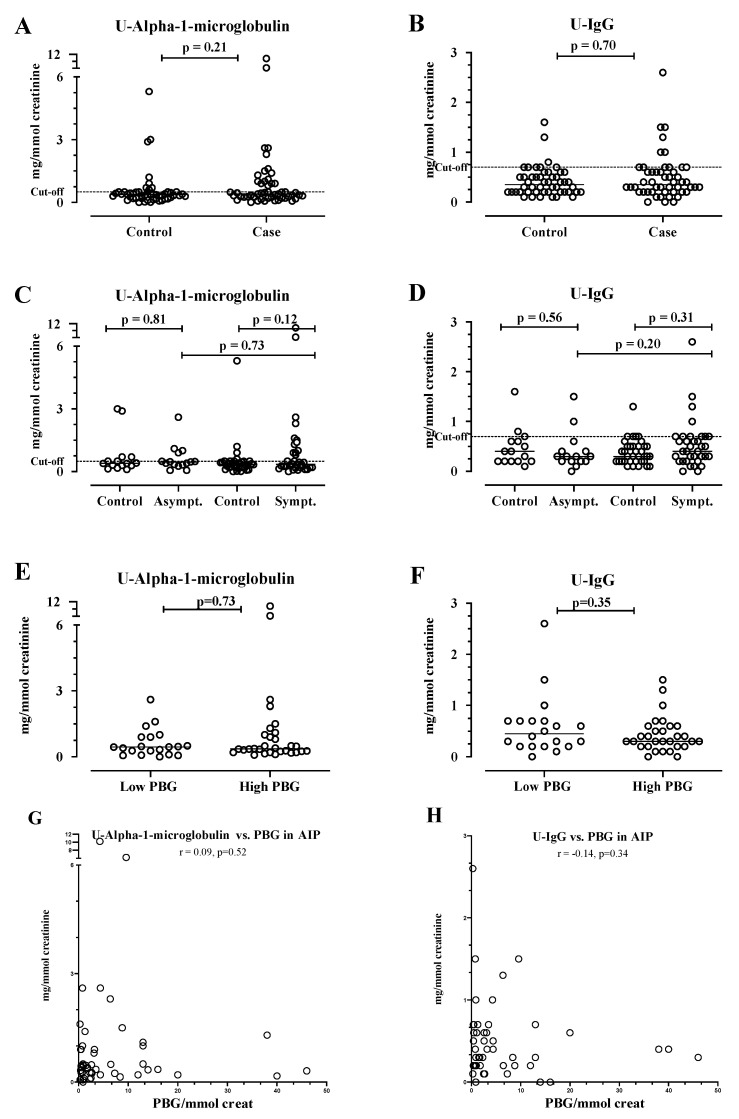
(**A**,**B**) Urine alpha-1-microglobulin and urine IgG in the AIP cases versus the matched controls, (**C**) in the symptomatic AIP cases versus the matched controls, (**D**) in the asymptomatic AIP cases versus the matched controls, and (**E**,**F**) in the AIP cases with high versus low PBG levels. (**G**) Correlation between urine PBG and urine alpha-1-microglobulin in the AIP cases and (**H**) between urine PBG and urine IgG in the AIP cases. In (**C**), a horizontal line corresponds to 0.7 mg/mmol creatinine, which is the cut-off for elevated versus low or normal urine alpha-1-macroglobulin. In (**D**), a horizontal dotted line corresponds to 0.8 mg/mmol creatinine, which is the cut-off for elevated versus low or normal urine IgG. All urine measurements are corrected for urine creatinine. U, urine; P, plasma.

**Figure A8:**
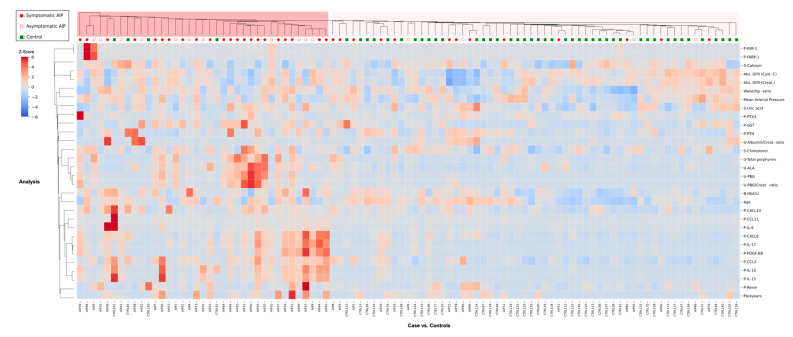
Correlation between plasma KIM-1, FABP-1, renin, and α-GST and porphyrins, porphyrin pre-cursors, cytokines, and GFR in the AIP cases. The figure represents a heatmap of mixed data. The rows represent the different analyses, while the columns represent the individual samples catego-rized as either AIP cases or controls. The heatmap was generated using single linkage and cosine distance metrics. The expression values are scaled as Z-scores and displayed using a color scale. The figure was generated using Python and was further edited in Adobe Illustrator. The script is available as Supplementary Information.

Urine Alpha-1 microglobulin and urine IgG were not significantly different between the AIP cases and the matched controls (*p* = 0.21 and *p* = 0.70, respectively, Figure A4, Appendix A). Similarly, no differences were found between the symptomatic AIP cases versus the asymptomatic AIP cases (*p* = 0.73 and *p* = 0.20, respectively, Figure A4, Appendix A) nor between the AIP cases with high PBG versus the AIP cases with low PBG (*p* = 0.73 and *p* = 0.35, respectively, Figure A4, Appendix A). Both urine Alpha-1 microglobulin and urine IgG did not correlate significantly with urine PBG in the AIP cases (ρ = 0.09, *p* = 0.52 and ρ = −0.14, *p* = 0.34, respectively, Figure A4, Appendix A).

### 3.9. Correlations among the Liver Marker Plasma Glutathione S-Transferase Alpha, the Plasma Kidney Markers Fatty Acid-Binding Protein-1, Renin, and Kidney Injury Molecule, and Conventional Kidney and Liver Markers in the Plasma and Urine in the AIP Cases

**Figure 9:**
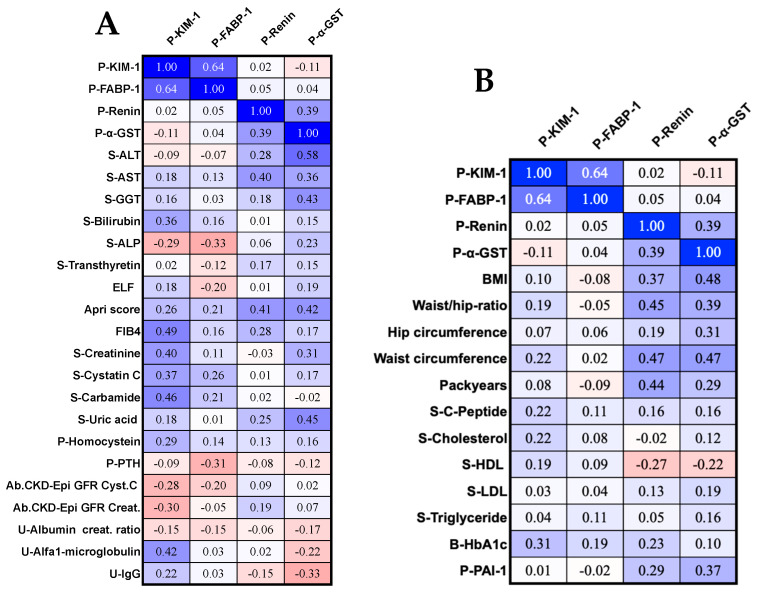
Correlation matrixes for novel kidney and liver markers. (**A**) Correlation matrixes of novel kidney and liver markers with standard measures of kidney and liver function in acute intermittent porphyria (AIP) cases (*n* = 50). (**B**) Correlation matrixes of kidney and liver markers, waist/hip ratio, and fat and glucose markers in the AIP cases (*n* = 50). The figure shows the ρ-values. The ρ-values ≤ −0.28 and ρ ≥ 0.28 correspond to a statistically significant *p*-value < 0.05. The ρ-values are colored on a scale from deep blue if +1 to bright red if −1, with white being zero. PAI-1, plasminogen activator inhibitor-1; KIM-1, kidney injury molecule-1; FABP-1, fatty acid-binding protein-1, α-GST, alpha-glutathione S-transferase; U, urine; P, plasma; S, serum; B, blood.

FABP-1 correlated significantly and negatively with parathyroid hormone in the plasma (*p* < 0.05) and with serum ALP (*p* < 0.05, Figure 9A). Plasma renin correlated significantly and positively with the APRI score and serum AST (both *p* < 0.05, Figure 9). Plasma α-GST correlated significantly and positively with serum ALT, AST, and GGT, the APRI score, and serum creatinine and uric acid and negatively with urine IgG (*p* < 0.05 for all, Figure 9A). Plasma KIM correlated significantly and positively with serum bilirubin, liver fibrosis score-4 (FIB-4), serum carbamide, plasma homocysteine, and urine alpha-1-microglobulin and significantly and negatively with the absolute GFR calculated using creatinine (CKD-EPI GFR equation, *p* < 0.05 for all, Figure 9A). Plasma KIM-1 correlated significantly and positively with the serum creatinine and serum cystatin C and significantly and negatively with the absolute GFR calculated using cystatin C (CKD-EPI GFR equation, *p* < 0.05 for all, Figure 9A, illustrated in Figure A3, Appendix A). Furthermore, the novel markers correlated with each other. Plasma KIM-1 correlated significantly and positively with plasma FABP-1 (*p* < 0.05), while plasma renin correlated significantly and positively with plasma α-GST (*p* < 0.05, Figure 9A).

### 3.10. Correlations among the Liver Marker Plasma Glutathione S-Transferase Alpha, the Plasma Kidney Markers Fatty Acid-Binding Protein, Renin, and Kidney Injury Molecule, Anthropometric Measures, and Smoking, Fat, and Glucose Markers in AIP

Plasma KIM-1 correlated significantly and positively with HbA1c (*p* < 0.05). Plasma renin correlated significantly and positively with BMI, waist/hip ratio, packyears, and plasma PAI-1 (*p* < 0.05). Plasma α-GST correlated significantly and positively with BMI, waist/hip ratio, pack–years, and plasma PAI-1 (*p* < 0.05, Figure 9B).

## 4. Discussion

In our study involving AIP patients and matched controls, we analyzed nine novel urine and four plasma markers that detect kidney and/or liver injury. Based on the known test characteristics of these novel markers characterized in Table A1, our findings indicate that AIP is associated with both proximal tubular kidney damage and hepatocyte damage. Notably, while some of these markers, like KIM-1, have FDA qualification for clinical research, they are not yet approved or available for clinical use. We found no differences in the absolute GFR as measured by traditional kidney markers, serum creatinine and cystatin C, between the AIP cases and the matched controls. We have previously reported no difference in eGFR between AIP cases versus matched controls, although differences were observed in subgroups [6]. As for liver markers, serum AST, FIB-4, and ELF were similar in the AIP cases versus the controls in this study, while a slightly higher serum ALT was observed in the AIP cases, which has been previously reported [6]. Hence, our data suggest that these novel kidney and liver markers, plasma KIM-1, plasma FABP–1, urine TIMP–1, urine FABP-1, and plasma α-GST, may detect early-stage damage during a phase of kidney and liver stress before standard kidney and liver markers are abnormal.

Despite no marked differences in conventional kidney parameters such as absolute GFR (measured by serum creatinine and cystatin C) between the AIP cases and controls, the plasma levels of KIM-1 were significantly elevated in the AIP cases in this study. Because KIM-1 is an indicator of proximal tubular damage, this result corroborates the findings from Pallet et al. who found evidence of proximal tubular damage in AIP [28]. KIM-1 may enhance the sensitivity of the detection of kidney damage, potentially averting permanent injury in AIP patients. KIM-1 is barely detectable in healthy kidneys [29], and a significant increase in KIM-1 expression in the proximal tubuli has been found both in acute kidney injury (AKI) and chronic kidney disease (CKD) [29]. A prospective study of 549 individuals with biopsy-confirmed kidney disease involved the analysis of 225 circulating plasma proteins and found that KIM-1 was the top-performing marker positively associated with acute tubular injury [30]. It is significantly activated in response to ischemic and toxic damage to the kidney [29,31,32,33].

Interestingly, in the present study, urine PBG correlated significantly and positively with plasma KIM-1 levels, indicating that PBG is associated with KIM-1 and potentially mechanistically involved in the development of chronic kidney disease in AIP. These findings are in line with a large study of acute hepatic porphyria that observed an increased risk of CKD in AIP, particularly in those with elevated PBG [4]. While a previous study observed significantly higher PBG in AIP cases with eGFR < 60 compared to AIP cases with eGFR > 60, no significant differences in ALA levels were observed between the eGFR groups [2]. This is in line with our finding that urinary ALA did not correlate significantly with KIM-1.

In prior research, we demonstrated low-grade inflammation in AIP as measured by elevated plasma cytokines compared to controls [7]. We found that relative eGFR based on cystatin C and the inflammatory marker long pentraxin-3 were significant predictors of AIP disease activity, as measured by PBG in the urine [7]. In the current study, when comparing the cytokine levels in the AIP cases with elevated cystatin C to those with normal cystatin C levels, we observed significantly higher levels of CCL2 and CXCL10 in the group with elevated cystatin C. Additionally, the plasma cytokines CXCL10 and CCL2 (monocyte chemotactic peptide) were positively correlated with plasma KIM-1, suggesting an association between CCL2, CXCL10, and kidney disease in AIP. CCL2 is produced by renal cells and plays a role in acute ischemic and toxic kidney injuries [34]. It is proposed as a biomarker for kidney fibrosis and kidney function decline [35]. Moreover, chronic KIM-1 expression in a mouse model resulted in inflammation, tubule interstitial fibrosis, elevated CCL2 levels, and increased CCL2-dependent macrophage chemotaxis [36].

Because plasma KIM-1 is a relatively new biomarker, we wanted to explore its relationship with other markers in AIP. As expected, it correlated significantly and positively with serum creatinine, cystatin C, carbamide, and urine alpha-1 microglobulin, which are all surrogate markers for renal function. Surprisingly, plasma KIM-1 correlated positively and significantly with the Liver Fibrosis Index FIB-4, but this finding could fit with a recent report describing associations between an FIB-4 score above 1.3 and incident CKD [37]. Furthermore, we found a significant correlation between the intake of vegetables in grams and KIM-1. While we suspect that this finding might be due to random variation, one case study reported acute tubular kidney failure in a person with excessive intake of oxalate-containing vegetables and berries [38]. We found significant positive correlations between age and plasma KIM-1 and significantly negative correlations between age and absolute GFR based on the cystatin C equation. This is consistent with another AIP study that measured eGFR ten years apart and showed a decline in GFR of 1 mL/min per 1.73 m^2^ each year in symptomatic AIP patients [2].

Notably, while plasma KIM-1 was significantly elevated in the AIP cases, urine KIM-1, which is also a marker of proximal tubule injury, was not [39,40]. Furthermore, while urine PBG did correlate significantly with plasma KIM-1, it did not correlate significantly with urine KIM-1. KIM-1 is not only expressed in proximal tubular cells but also in microglial cells, excitatory neurons, oligodendrocytes, and inhibitory neurons [31,32]. This implies that our findings of elevated plasma KIM-1 could potentially have other cellular sources than proximal tubular cells. Additionally, it has been suggested that while urine KIM-1 reflects acute protein production, which can vary substantially over time, plasma KIM-1 may serve as a marker of chronic protein production; therefore, plasma KIM-1 might be a more reliable marker of chronic rather than acute injury [41,42]. Furthermore, these two markers may mirror distinct facets of proximal tubular damage [41,42]. Specifically, urine KIM-1 may signify the extent of damage within the proximal tubule, whereas plasma KIM-1 could signify the loss of tubular cell polarity during injury [41,42].

Studies have emphasized the relationship between KIM-1 and specific renal diseases, such as renal transplant rejection [43], cisplatin-associated AKI [44], the risk of chronic kidney disease after cardiac surgery [45], or the risk of acute renal failure in patients with acute coronary syndrome or heart failure undergoing coronary angiography [46]. However, none of the studies conclude on the role of novel markers in clinical practice. The epidemiological study by Schulz et al. indicates that elevated KIM-1 may indicate a more rapid decline in renal function [42].

The significantly elevated levels of urine TIMP-1 in the symptomatic AIP cases versus the controls provide evidence of proximal tubular damage in AIP. Tissue inhibitors of metalloproteinases (TIMP-1) is an inhibitor of matrix metalloproteinases MMP-1-3 and MMP-7-9 [47,48]. MMPs can degrade and remodel proteins in the extracellular matrix [48]. In this way, TIMP-1 may participate in the promotion of tubulointerstitial fibrosis by inhibiting the MMPs in the kidneys and worsening inflammation and kidney scarring [49]. A study of children reported that elevated levels of urine TIMP-1 collected after admission to a pediatric intensive care unit might be an independent predictor of acute kidney injury and mortality [49]. The lack of a correlation between TIMP-1 with porphyrin precursors in this study suggests that the mechanism for increased urine TIMP-1 levels may be independent of porphyrin precursors in AIP. Other potential causes for damage to the proximal tubule in AIP range from medication side effects to factors like ischemia and hypoxia [50]. Notably, certain drugs, including Normosang used during acute porphyria attacks, have potential renal side effects.

Urine renin was elevated in the symptomatic AIP cases compared with the matched controls, but we did not see differences in plasma renin. Renin originates in the circulation and is filtered into the urine, with 90% being reabsorbed into the proximal tubules. However, if proximal tubular damage is present, less will be reabsorbed, and urine renin levels will rise [28]. The latter may explain our finding of significantly elevated urine renin in the symptomatic AIP cases versus the matched controls.

Because urine collagen IV correlated significantly and positively with ALA and total porphyrins and coproporphyrins, we cannot rule out the possibility that porphyrins and porphyrin precursors damage the glomerular basal membrane. In previous studies, elevated levels of collagen IV in urine were associated with reduced glomerular function in diabetic nephropathy, perhaps due to the thickening of the glomerular basement membranes [51]. Despite correlations with ALA, total porphyrins, and coproporphyrins, because we found no significant differences in urine collagen IV between the AIP cases and matched controls or between the symptomatic AIP cases and the controls, we therefore speculate that the glomerular basal membrane is not affected in AIP.

The urine markers calbindin, TFF-3, CXCL10, albumin/creatinine, and IgG demonstrated no significant differences, and, as such, do not seem promising as early markers of AIP-related kidney damage.

We observed elevated plasma levels of the liver and kidney injury marker FABP-1 in the AIP cases compared with the matched controls and significantly higher plasma FABP-1 and urine FABP-1 in the high PBG group, suggesting that PBG may be involved in the mechanism of FABP-1 elevation. Other potential mechanisms of FABP-1 elevation, such as elevated lipids, starvation, and a high-carbohydrate diet, which are all known to have effects on FABP-1 [52], could potentially be contributing. The levels of obesity and diabetes were the same between both study groups [7]. Notably, FABP-1 levels are known to be elevated in HCC [53], a known complication of AIP [11], and FABP-1 is a sensitive marker for the detection of hepatocellular damage [53]. Although our study did not involve AIP patients under HCC treatment, one might surmise that the AIP patients with the highest plasma FABP-1 may be the ones at greatest risk of HCC. This topic deserves additional research to understand the significance of FABP-1 for HCC in AIP. Plasma FABP-1 is also a marker of proximal tubular injury because it is filtered freely by the glomeruli and reabsorbed by the proximal tubule [33,54]. However, the heightened plasma FABP-1 not mirrored by urine FABP-1 in our study suggests liver damage rather than renal damage. This liver damage, if present, does not seem to be associated with fibrosis, as the fibrosis markers (ELF score and FIB-4) were similar in the AIP cases versus the matched controls. This is consistent with the fact that HCC in AIP typically emerges without cirrhosis [55,56].

In the plasma of the AIP cases versus the controls, we found significantly higher levels of α-GST (p < 0.001), which is a marker of hepatocellular injury, indicating that this may be a sensitive marker of liver injury in AIP. Injury to hepatocytes and low-grade cytolysis led to the release of α-GST into the blood stream since it was highly correlated with serum ALT. The elevation of α-GST in AIP is consistent with our previous finding of slightly elevated serum ALT in AIP patients versus matched controls (p < 0.05). Moreover, previous studies have shown that α-GST is overexpressed in HCC [57]. α-GST is known to negatively regulate the mTOR signaling pathway, which is thought to be involved in protection against HCC [57]. Consequently, α-GST may be another marker that could identify patients at risk of HCC. We found no difference in urine α-GST among the groups. While urine α-GST is a marker of acute kidney injury, specifically renal tubular leakage, it did not show good test performance in a multicenter study of 252 adults undergoing cardiopulmonary bypass [58]. Therefore, the ability to interpret this biomarker in our cohort is limited.

Our findings can hopefully promote further research with the aim of better understanding kidney disease, liver disease, and HCC in AIP. In the future, the measurement of specific serum and urine biomarkers may allow for the separation of disease phenotypes in AIP. Furthermore, these sensitive and early markers of organ damage may be valuable for monitoring for AIP-related organ damage, both for patients on and off treatment [59].

This study has several strengths. Firstly, it delivers a unique and comprehensive examination of modern kidney and liver damage markers within the context of AIP, establishing correlations with various biochemical markers. Secondly, the study benefits from a substantial participant cohort, an achievement of significance given the rarity of the disease. Nonetheless, certain limitations warrant consideration. Primarily, the study did not have access to biopsy samples or ultrasound of the kidney or liver to provide verification of the data. Also, in this case–control study, all samples were collected at a single time point for each patient, and the present study therefore lacks the capability to determine whether these biomarkers can predict kidney or liver damage in AIP. Additionally, the plasma and urine samples used for analyzing modern liver and kidney markers had been stored for 10 years in a freezer at −80 °C. Therefore, we cannot completely rule out the possibility that the samples could have deteriorated slightly [60], potentially influencing the results to some extent. Regardless, if such an unexpected change had occurred, it would have affected both the cases and the controls equally. Also, we cannot rule out that the AIP patients may have other undiagnosed conditions that are responsible for the changes in kidney and liver biomarkers. It is pertinent to recognize that most participants shared a common pathogenic AIP variant and all are from Norway. Although this could impact the generalizability of the findings, it is imperative to acknowledge that this pathogenic variant is prevalent in both Sweden and Norway, and all pathogenic AIP variants uniformly manifest with diminished HMBS enzyme function.

## 5. Conclusions

This study assessed biomarkers to explore sensitive and early markers of kidney and liver injury in AIP patients versus matched controls. Elevated plasma levels of the kidney marker KIM-1, the liver marker α-GST, and the kidney and liver marker FABP-1 in AIP cases suggest proximal tubular kidney damage and hepatocellular damage. Furthermore, plasma KIM-1 demonstrated a significant positive correlation with the AIP disease activity marker urine PBG and other inflammatory markers like P-CXCL10, CCL2, and TCC. Similarly, AIP patients with high PBG levels had increased FABP-1 in both their plasma and urine. The observed association between porphyrin precursors and biomarkers for kidney and liver stress warrants further in-depth and longitudinal studies encompassing genetic variation and biopsy results.

## Figures and Tables

**Table 1:** Baseline demographic characteristics of the study population.

	Controls	AIP ^a^ Cases	*p*	Reference Values
Age, years (M ± SD)	50.4 ± 18.6	50.6 ± 18.6	0.38	
Woman	21 (42%)	21 (42%)		
Men	29 (58%)	29 (58%)		
Symptomatic AIP	0	35 (70%)		
Asymptomatic AIP	0	15 (30%)		
Recurrent acute porphyria *	0	2 (4%)		
Sporadic acute porphyria *	0	20 (40%)		
Asymptomatic acute porphyria *	0	7 (14%)		
Symptomatic high excreter *	0	3 (6%)		
Asymptomatic high excreter *	0	6 (12%)		
Latent porphyria *	0	12 (24%)		
Body mass index, kg/m^2^ (SD)	27.2 (3.9)	27.2 (3.9)	0.92	20–25 ^c^
U-PBG/creatinine, µmol/mmol/creatinine ^a^	0.4 (0.3–0.5)	2.6 (0.9–8.5)	<0.0001	<1.5
U-ALA/creatinine, µmol/mmol/creatinine ^a^	1.9 (1.6–2.3)	3.9 (2.4–7.0)	<0.0001	<3.9
Mean arterial pressure (MAP), mmHg	89 (83–97)	89 (81–97)	0.69	70–100
S-creatinine, µmol/L	77 (70–87)	83 (72–88)	0.29	<90, <105 ^d^
Absolute CKD-EPI GFR, Cystatin C eq., mL/min	94 (77–110)	98 (67–112)	0.65	
S-Aspartate aminotransferase (S-AST), U/L	21 (18–26)	24 (19–29)	0.17	<35, < 45 ^e^
APRI score	0.19 (0.14–0.22)	0.21 (0.16–0.33)	0.049	<0.77 ^f^
FIB-4	0.75 (0.47–1.2)	0.83 (0.50–1.4)	0.25	<1.3
ELF score	8.6 (7.9–9.4)	8.5 (7.9–9.3)	0.93	6.7–9.8 ^g^
<60 mL/min/1.73 m^2^	3 (6%)	9 (18%)	0.12	
Moderately increased, high, very high CKD risk ^b^	7 (14%)	11 (22%)	0.44	
Diabetes mellitus n (%)	3 (6%)	4 (8%)	1.0	
Hypertension, syst. BP ^h^ ≥ 130 or diast. ≥ 80, n (%)	18 (36%)	18 (36%)	1.0	
Hypertension, syst. BP ^h^ ≥ 140 or diast. ≥ 90, n (%)	6 (12%)	7 (14%)	> 0.99	
Overweight, body mass index, 25–29.9 kg/m^2^, n (%)	19 (38%)	26 (52%)	0.23	
Obesity, body mass index, ≥ 30 kg/m^2^, n (%)	15 (30%)	12 (24%)	0.65	
^i^ Alcohol intake > 30 g/day, n (%)	3 (6.5%)	4 (8.7%)	> 0.99	

The data represent the mean ± standard deviation (M ± SD), n (%), or median values and the IQR from 50 acute intermittent porphyria (AIP) cases and 50 matched controls. Symptomatic AIP was defined as ever having an AIP attack. Asymptomatic AIP was defined as having pathogenic variants associated with AIP but never having experienced an AIP attack. The Wilcoxon signed-rank test for matched pairs was used for continuous, matched variables, and Fisher’s exact test was used for categorical variables. The *p*-values are exact two-tailed *p*. * New consensus-based definitions explained in Stein et al. [16]. ^a^ Acute intermittent porphyria (AIP) disease activity markers: PBG, porphobilinogen; ALA, delta-aminolevulinic acid. ^b^ Prognosis of CKD according to GFR and albuminuria: low risk, moderately increased risk, high, and very high risk as defined by kdigo.org 2012 [22]. ^c^ Normal body mass index, BMI, for persons above 70 years: 22–27. ^d^ The reference value for S-creatinine for women is <90 µmol/L and for men, it is <105 µmol/L. ^e^ The reference value for S-AST for women is <35 U/L and for men, it is <45 Ul/L, ^f^ Cut-off for the APRI score for significant fibrosis > 0.77, severe fibrosis > 0.80, and cirrhosis > 0.83, ^g^ The reference value for ELF is 6.7 to 9.8, but for men, it is 7.0–9.9, and for women, it is 6.6–9.3, and for the high-sensitivity exclusion of fibrosis, it is 7.7., and for the high-specificity identification of fibrosis, it is 9.8. U, urine; S, serum. ^h^ BP, blood pressure in mmHg; syst., systolic; diast., diastolic, ^i^ Alcohol intake in g/day from a 7-day diet logbook described previously [27].

**Table 2:** Subgroups of AIP cases with high and normal S-cystatin C compared to cytokines and eGFR.

	Cases with High Cystatin C *n* = 16	Cases with Normal Cystatin C *n* = 34	*p*-Value Cases high vs. Normal Cystatin C	Matched Controls for Cases with High Cystatin C *n* = 16	*p*-Value for Cases with High Cystatin C vs. Matched Controls	Matched Controls for Cases with Normal Cystatin C *n* = 34	*p*-Value for Cases with Normal Cystatin C vs. Matched Controls
Cyst. C	1.3 (1.1–1.6)	0.9 (0.8–1.0)	<0.0001	0.98 (0.92–0.99)	<0.0001	0.95 (0.84–1.1)	0.008
eGFR	52 (38–79)	94 (76–109)	<0.0001	83 (70–93)	<0.0001	90 (68–101)	0.009
IL-1β	1.6 (1.1–3.1)	2.0 (0.9–4.0)	0.99	0.92 (0.79–1.2)	0.01	0.86 (0.65–1.2)	0.0003
IL-6	5.0 (2.3–9.0)	5.0 (2.0–11)	0.80	2.0 (2.0–3.0)	0.03	2.0 (0.61–3.0)	0.0006
CXCL8	8.5 (5.3–14)	8.0 (6.0–15)	0.85	5.0 (3.3–6.8)	0.02	5.0 (3.0–7.3)	0.0005
CXCL10	1072 (969–1593)	850 (657–1112)	0.02	603 (376–879)	0.004	601 (402–962)	0.03
CCL2	21 (15–25)	14 (12–20)	0.04	12 (8.3–14.0)	0.007	10 (8.0–13)	0.0002

The data represent the median values and IQR from 16 acute intermittent porphyria (AIP) cases with high serum cystatin C and their 16 matched controls and 34 AIP cases with normal serum cystatin C and their 34 matched controls. The Mann–Whitney U test was used for cases (AIP cases) with high vs. normal cystatin C. The Wilcoxon matched-pairs signed-rank test was used for cases with high cystatin C vs. matched controls and cases with normal cystatin C vs. matched controls. High cystatin C was defined as levels above the reference level. Reference level < 0.97 for 1–50 years and <1.25 for >50 years. S-cystatin C in mg/L, eGFR = Relative eGFR CKD-EPI, mL/min/1.73 m^2^, based on the cystatin C equation. All cytokines in the plasma are in pg/mL.

**Figure A5:**
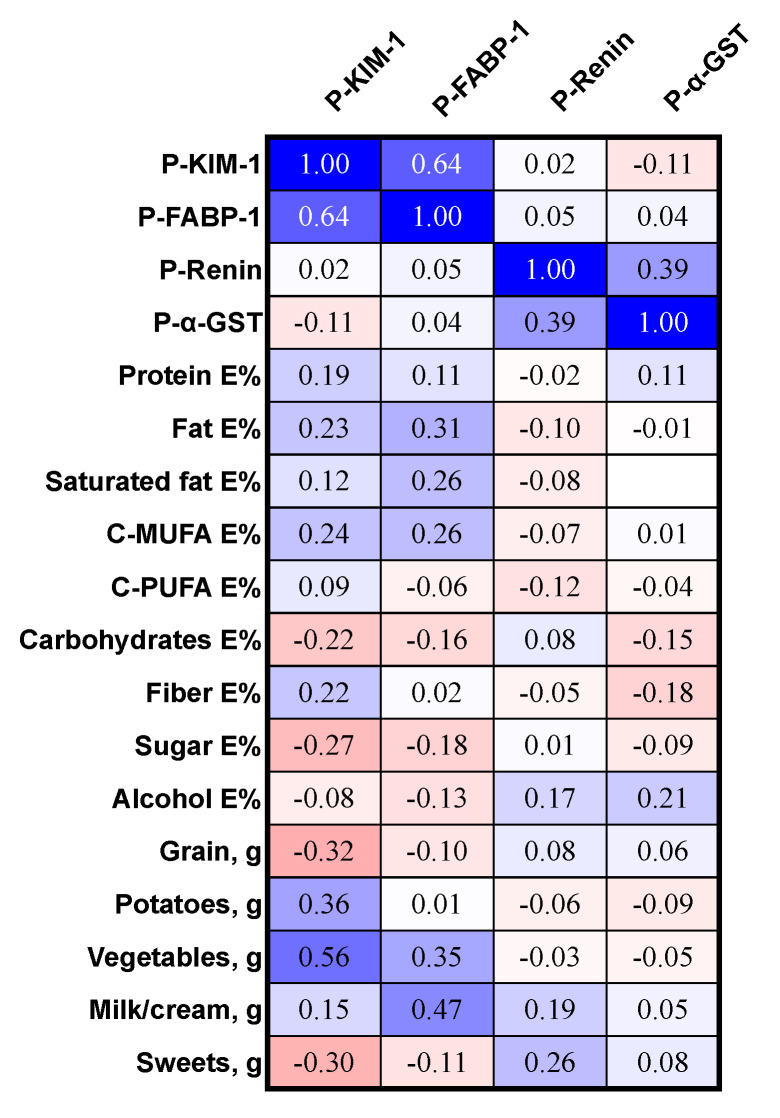
Correlation among plasma KIM-1, FABP-1, renin, and α-GST and dietary markers in the AIP cases. Correlation matrix of dietary intake in percent of energy intake (E%) and g per day versus plasma kidney injury molecule-1 (KIM-1), fatty acid-binding protein-1 (FABP-1), renin, and glutathione S-transferase alpha (α-GST) in the acute intermittent porphyria group (*n* = 47). The table shows ρ-values. The ρ-values ≤ 0.28 and ρ ≥ 0.28 correspond to statistically significant *p*-value < 0.05. The ρ-values are colored on a scale from bright blue if +1 to bright red if −1, with white being zero.

**Figure A6:**
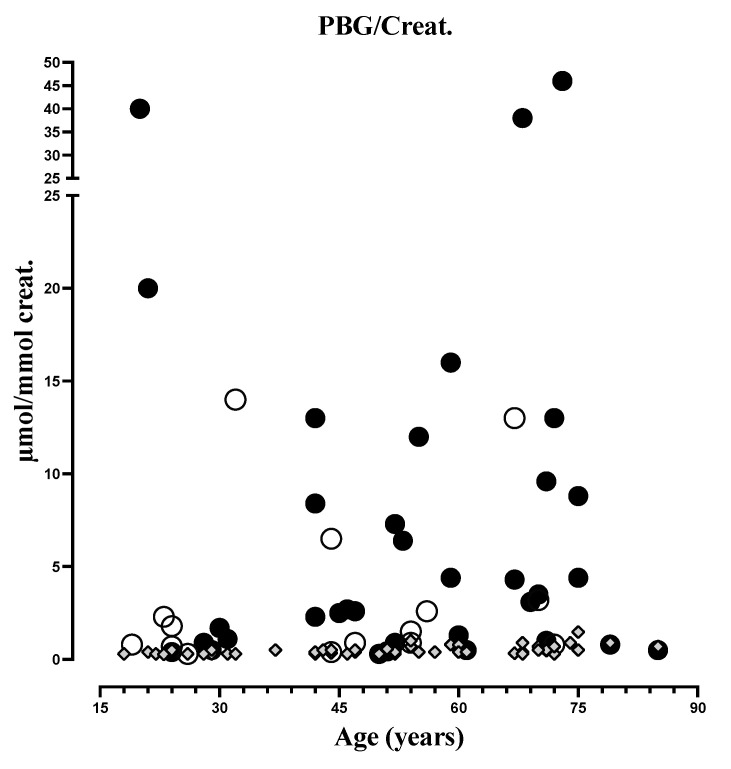
Correlation between urine PBG/creatinine ratio (µmol/mmol/creatinine) and age in years in symptomatic AIP cases (filled black dots), asymptomatic AIP cases (open circles), and controls (grey diamonds). Each person is represented by a single PBG measurement outside AIP attacks. A prior study examining the biological variation of urine ALA and urine PBG in AIP implied that patients with AIP exhibit variations regarding their respective typical porphyrin precursor levels [72].

**Figure A7:**
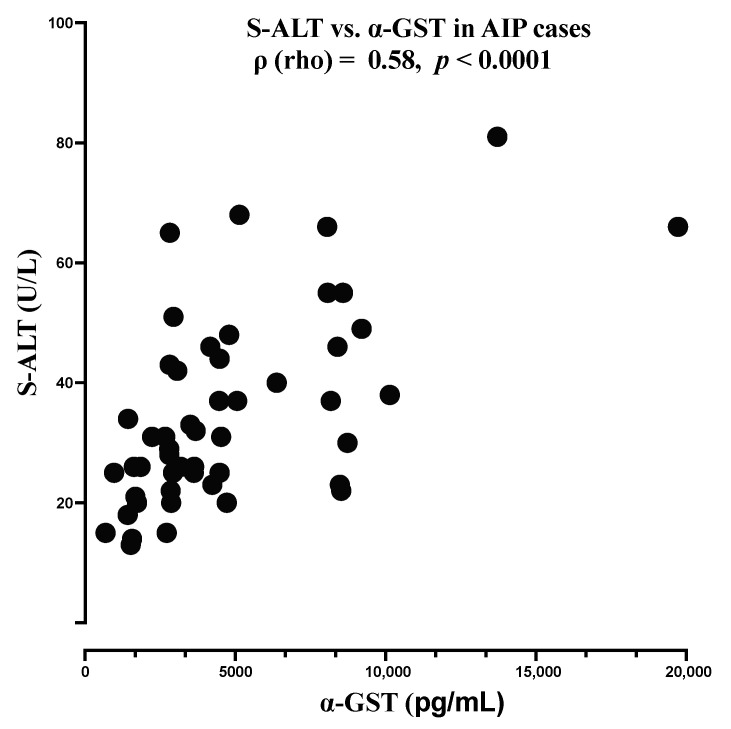
Correlation between S-ALT (U/L) and α-GST in plasma (pg/mL) in the AIP cases (filled black dots).

## Data Availability

Data supporting reported results can be obtained upon request.

## Supplementary Materials

The following are available online at: https://www.mdpi.com/2075-1729/14/1/19/s1. Table S1: STROBE statement-checklist. Table S2: The code for clustering performed in Figure A8.

## Appendix A

**Table A1:** Overview of kidney and liver markers.

Analyte	Probable Site of Damage or Decreased Function
P-KIM-1 *^#&^ and U-KIM-1	Proximal Tubular Damage, 38.7 kDa (34–44), transmembrane glycoprotein type-1, also activated in also response to ischemic and toxic kidney damage [29,31,32,33]. Expressed in: proximal tubular cells, microglial cells, excitatory neurons, oligodendrocytes, and inhibitory neurons [31,32]. Markers of proximal tubular cell injury in both plasma and urine [39,40]. KIM-1 has anti-inflammatory properties and clears apoptotic cells [33]. Other name: hepatitis A virus cellular receptor-1 *
P-FABP-1 *^^^ and U-FABP-1 ^^&^	Proximal Tubular Damage, but also a marker in hepatocellular carcinoma, 14 kDa, soluble protein found in hepatocyte cytoplasm and other tissues, notably kidney tubular cells and mitochondria. FABP-1 is expressed in enterocytes, hepatocytes, Paneth cells, enteroendocrine cells, and proximal tubular cells [31,32]. Central to lipid metabolism, oxidative stress protection, heme transport, and metabolic disorders like diabetes, cancer, and obesity [52]. Other name: liver FABP.
U-TIMP-1 ^ # ^	Proximal Tubular Damage, 11–23 kDa, expressed in: mesothelial cells, monocytes, Langerhans cells, and fibroblasts; renal cancer prognostic marker [31,32]. Biomarker for complications of metabolic diseases including diabetic decompensation, fatty liver disease, and diabetic nephropathy [48].
U-*α-*GST	Proximal Tubular Damage, potentially distal tubular involvement as well, 25 kDa. After renal injury, U-α-GST is found in the proximal tubular cells [61] and elevated levels in the urine suggest epithelial necrosis of the proximal tubules [61].
P-*α-*GST * ^ # ^	Hepatocellular Injury, cytosolic GSTs are composed of two subunits which are 25 kDa each [57]. Detoxification enzyme, cell signaling, chemotherapeutic drug resistance, and post-translational modifications [57]. The enzyme α-gluthatione S-transferase constitutes around 3% of the hepatocyte’s cytosolic protein [62]. *α-*GST i*s* distributed uniformly in the liver lobule, while AST and ALT are found mainly in the periportal hepatocytes [62].
U-Calbindin	Distal tubular damage, 28 kDa, expressed in distal tubules, also in the collecting ducts, intestines, brain, and pancreas [63].
U-Collagen IV	Glomerular damage. The glomerular basal membrane is a highly structured matrix of type IV collagen and laminins, nidogen, and sulfated proteoglycans [51,64]. Elevated levels of collagen IV in the urine are associated with reduced glomerular function in diabetic nephropathy and membranous nephropathy, perhaps due to thickening of the membranes [51]. Other kidney diseases where collagen IV is involved are Alport’s disease due to mutations and Goodpasture´s syndrome caused by immune attack [64] which causes disruption of the attached epithelia and thereby collagen exposure and hence organ impairment [64].
U-TFF-3	Various kidney tissues, potential CKD marker, conflicting evidence on drug-related nephrotoxicity [65,66]. A small peptide hormone from epithelial cells in the gastrointestinal tractus which takes part in the repair of the epithelial surfaces there [65]. Also produced in cells in the collecting ducts in the kidney, where its function is uncertain, but it might be related to ongoing kidney repair [65].
P-Renin and U-Renin	Juxtaglomerular apparatus, glomerular, and tubular, 48 kDa [28]. Renin is a specific endopeptidase that is secreted from juxtaglomerular cells that surround the afferent arterioles where they enter the glomerulus [67]. Blood pressure regulation.
U-CXCL10	Chemokine, inflammation. CXCL10 plays a role in immune responses and inflammation [68]. In a study in which pediatric kidney transplant experts participated, the impact of urinary CXCL10/creatinine on the perception of the risk of T-cell mediated rejection in organ transplant recipients was evaluated. It was found that use of U-CXCL10/creatinine improved the probability estimates [69].
Conventional markers:	
U-α-1-microglobulin	α-1-microglobulin is produced in the liver. It is filtered unhindered in the normal glomeruli and is reabsorbed and broken down in the proximal tubuli. It is a biomarker for proximal tubular damage [39].
U-Albumin	Albumin, 67 kDa, is elevated in the urine during glomerular kidney damage. It is also associated with mortality and cardiovascular disease [70]. U-Albumin/creatinine-ratio together with eGFR is widely used to stage kidney disease [71].
Urine-IgG	Urine-IgG is a protein of approximately 150 kDa. To divide between selective and unselective glomerular damage, U-IgG together with U-albumin can be measured since they differ in size. If the relatively large IgG is found in the urine, this may indicate a non-selective glomerular damage.

P = plasma (P), U = urine (U), TIMP-1 = tissue inhibitors of metalloproteinases-(TIMP-1), kidney injury molecule-1 (KIM-1), α-glutathione S-transferase (α-GST), fatty acid-binding protein (FABP-1), chemokine-X-X-motif chemokine ligand 10 (CXCL10), Trefoil factor-3 (TFF-3). * = *p* < 0.05 between the AIP cases vs. the matched controls. ^#^ = *p* < 0.05 between the symptomatic AIP cases vs. the matched controls. ^^^ = *p* < 0.05 between the AIP cases with high PBG vs. the AIP cases with low PBG. ^&^ = correlates significantly and positively with PBG. Bold markup for significant differences between the AIP cases and controls or described subgroups.
