# Supplementary material for: Potential Biomarkers for the Earlier Diagnosis of Kidney and Liver Damage in Acute Intermittent Porphyria
Source: Life (Basel). 2023 Dec 21;14(1):19. doi: 10.3390/life14010019 (PMC11154556; doi:10.3390/life14010019)
Supplement: Supplementary file 1 [file life-14-00019-s001.zip › Supplementary Table S1 STROBE statement-checklist.pdf]

STROBE Statement—checklist of items that should be included in reports of observational studies

	Item No.	Recommendation	Page No.	Relevant text from manuscript
**Title and abstract**	1	(*a*) Indicate the study's design with a commonly used term in the title or the abstract	1	Urine PBG testing and kidney and liver injury panels, including both routine and modern biomarkers, were performed on plasma samples from 50 AIP cases and 50 matched controls and urine samples from 48 matched pairs.
		(*b*) Provide in the abstract an informative and balanced summary of what was done and what was found	1	This study explored differences in modern biomarkers for renal and hepatic damage between AIP patients and controls. Urine PBG testing and kidney and liver injury panels, including both routine and modern biomarkers, were performed on plasma samples from 50 AIP cases and 50 matched controls and urine samples from 48 matched pairs. In conclusion, KIM-1, FABP-1 and α-GST are potential early indicators of renal and hepatic damage in AIP and are associated with porphyrin precursors and inflammation.
**Introduction**				
Background/rationale	2	Explain the scientific background and rationale for the investigation being reported	2, 3	Porphyria-associated kidney disease (PAKD) is seen in more than half of the patients with symptomatic AIP [1-3], and more than half of patients with PAKD have hypertension [1]. CKD and overall kidney diseases have been shown to be more common in patients with acute

				porphyria, of which AIP is the most common type, compared to reference populations, and especially in AIP patients with elevated urine porphobilinogen (PBG) [4]. Porphyrin precursors may induce oxidative stress and mitochondrial dysfunction in the renal tubular cells, which is associated with tubular dysfunction in AIP [2]. For optimal prevention and treatment of AIP-related kidney and liver damage, early detection and understanding of the damage type are crucial. Traditional surrogate markers like serum creatinine and eGFR are insufficient for early kidney damage detection, and AST and ALT are late liver damage markers. It is well known that AIP cases have a high risk of hepatocellular cancer (HCC) [10,11]. Interestingly, HCC in AIP cases is not associated with liver fibrosis [12]. More targeted diagnostic strategies using specific and sensitive modern kidney and liver damage markers together with traditional markers could potentially improve patient outcomes.

Objectives	3	State specific objectives, including any prespecified hypotheses	3	Hence, our study aimed to identify specific and sensitive markers for kidney and liver damage in AIP patients, hypothesizing that distinctions in these markers between AIP cases and matched controls and among different AIP patient subgroups would be correlated with inflammatory markers and porphyrin precursors.
**Methods**				
Study design	4	Present key elements of study design early in the paper	3	We conducted a case-control study of 50 genetically confirmed AIP cases and 50 controls matched for age, sex, and place of residence. The inclusion period was from September to November 2012. Participants lived in the Norwegian counties Nordland, Troms, Trøndelag, and Oslo
Setting	5	Describe the setting, locations, and relevant dates, including periods of recruitment, exposure, follow-up, and data collection	3	We conducted a case-control study of 50 genetically confirmed AIP cases and 50 controls matched for age, sex, and place of residence. The inclusion period was from September to November 2012. Participants lived in the Norwegian counties Nordland, Troms, Trøndelag, and Oslo.
Participants	6	(*a*) *Case-control study*—Give the eligibility criteria, and the sources and methods of case ascertainment and control selection. Give the rationale for the choice of cases and controls	3	As a practically possible approach for measurements of

				modern kidney and liver markers of the rare disease AIP we included 50 AIP patients, of whom 35 were symptomatic (ever had an AIP attack), and 15 were asymptomatic (never had an AIP attack), and 50 matched controls. From the initial 50 AIP cases and 50 controls, there was an insufficient amount of urine samples available in the freezer for two of the AIP cases. As a result, these two AIP cases and their corresponding controls were omitted from the urine analysis for the modern kidney and liver biomarkers.
		(*b*) *Case-control study*—For matched studies, give matching criteria and the number of controls per case	3	We conducted a case-control study of 50 genetically confirmed AIP cases and 50 controls matched for age, sex, and place of residence
Variables	7	Clearly define all outcomes, exposures, predictors, potential confounders, and effect modifiers. Give diagnostic criteria, if applicable	4	The absolute glomerular filtration rate was calculated for each study participant using the Chronic Kidney Disease Epidemiology Collaboration (CKD-EPI) creatinine equation (mL/min) and the cystatin equation (mL/min), adjusting for body surface area by including each participants measured height and weight to the equation [18]. In addition, we calculated the estimated GFR (eGFR) using the creatinine equation (mL/min/1.73m^2), as it is used for categorizing risk for CKD as defined by kdigo.org 2012 [19]. The AST to platelet Ratio Index (APRI) [20], the liver fibrosis index 4 (FIB4) [21], and the

				enhanced liver fibrosis (ELF) [22] scores were calculated as previously described. APRI= (aspartate aminotransferase (AST)/upper limit of normal of (AST)) X 100/platelet count as 10^9/L)[20]. FIB4 = (age × AST)/(platelets × (sqr.(ALT)), with age in years, aspartate aminotransferase (AST) and alanine aminotransferase (ALT) in U/L, and platelet count in 10^9/L. ELF score = 2.278 + 0.851 ln (cHA) + 0.751 ln (cPIINP) + 0.394 ln (cTIMP-1), where HA= hyaluronic acid, PIINP = amino terminal propeptide of type III procollagen, TIMP-1 = tissue inhibitor of metalloproteinases, and c = concentration [22] . HA, PIINP and TIMP-1 for the ELF test were analyzed on the Advia Centaur immune assay system in 2015. .
Data sources/ measurement	8*	For each variable of interest, give sources of data and details of methods of assessment (measurement). Describe comparability of assessment methods if there is more than one group	4	Cytokines in EDTA plasma were examined using multiplex technology in a case-control study involving 50 AIP cases, as detailed in a prior investigation [6]. Additionally, assessments of plasma C-peptide and plasminogen activator inhibitor-1, reported in pg/mL, were conducted using the Bio-Plex 200 system from Bio-Rad, together with a Bioplex Pro human diabetes immunoassay kit. Quality control measures were implemented during the assay procedures. The kidney markers in urine were analyzed

				with the 9-plex Human Kidney Injury Magnetic bead panel 1 from Merck KGaA (Darmstadt, Germany) on a Luminex® 200 system, and results were given initially as ng/mL. This urine panel consisted of nine markers: collagen IV, tissue inhibitors of metalloproteinases-1 (TIMP-1), kidney injury molecule-1 (KIM-1), α-glutathione S-transferase (α-GST), fatty acid binding protein (FABP-1), calbindin, chemokine-X-X-motif chemokine ligand 10 (CXCL10), trefoil factor-3 (TFF-3), and renin. All urine markers results were converted to picograms per milliliter (pg/mL) and corrected for urine creatinine (mmol) to compensate for differences in the concentration of urine with results expressed as pg/mmol creatinine.<br>The kidney markers in plasma in pg/mL were analyzed on a Luminex® 200 instrument system applying the 3-plex Human Kidney Injury Magnetic bead panel 4, Merck KGaA, catalogue number HKI4MAG-99K (P-KIM-1, P-Renin, and P-FABP-1) and the liver marker Human Liver Injury Magnetic bead panel from Merck KGaA, catalogue number HLINJMAG-75-K (P-α-GST). Though α-GST is a kidney damage marker in urine, it is a liver damage marker in plasma. The urine and plasma samples used for the new kidney markers had been stored frozen at -80°C from 2012 and were

				analyzed in 2021 (urine) and 2022 (plasma).<br>Measurements in urine of IgG and Alfa-1 microglobulin at Haukeland University Hospital in Bergen, and Albumin at Nordland Hospital in Bodø, were performed in 2012 with routine methods.
Bias	9	Describe any efforts to address potential sources of bias	5	Even when using the same assay kit and equipment, and the same sample handling, as we did, minor differences in the lower limit of detection (LLD) can be seen for reasons such as minor analytical variability and random chance since the analyses of samples regarding the modern liver and kidney markers on 50 AIP cases and 50 matched controls were performed in three different runs within a few days.
Study size	10	Explain how the study size was arrived at	3	As a practically possible approach for measurements of modern kidney and liver markers of the rare disease AIP we included 50 AIP patients, of whom 35 were symptomatic (ever had an AIP attack), and 15 were asymptomatic (never had an AIP attack), and 50 matched controls. From the initial 50 AIP cases and 50 controls, there was an insufficient amount of urine samples available in the freezer for two of the AIP cases. As a result, these two AIP cases and their corresponding controls were omitted from the urine

				analysis for the modern kidney and liver biomarkers.

Continued on next page

Quantitative variables	11	Explain how quantitative variables were handled in the analyses. If applicable, describe which groupings were chosen and why	5,6	The results were analyzed using GraphPad Prizm version 9 and 10 from GraphPad Software Inc. (San Diego, CA, USA). The cluster/heatmap figure (Appendix Figure A4) was generated using Python (Python 3.10.12 (main, Jun 11 2023, 05:26:28) [GCC 11.4.0] on Linux) and was further edited in Adobe Illustrator version 28 (64 bit), Adobe Inc., (San Jose, CA, USA). The heatmap was generated using single linkage and cosine distance metrics. The expression values are scaled as Z-scores, displayed using a color scale. The graphical abstract was made with BioRender.com
Statistical methods	12	(*a*) Describe all statistical methods, including those used to control for confounding	5	The Wilcoxon signed rank test for matched pairs was used for most of the data, comparing AIP cases versus matched controls. Fisher's exact test was used for categorical variables. When comparing a group of asymptomatic AIP cases versus a group of symptomatic AIP cases or AIP cases with high versus low PBG or ALA levels, the Mann-Whitney U-test was used. The Spearman's rank correlation coefficient was used for the AIP cases to calculate correlation coefficients (ρ) and two-tailed P-values. In the correlation matrixes, the color coding is deep blue for ρ-values approaching 1 and bright red for ρ-values of -1, while ρ values of 0.00 are white. Statistical significance was defined as $p < 0.05$.
		(*b*) Describe any methods used to examine subgroups and interactions	Page 6-16	See Figures 1-11 and Table 1 (including description)
		(*c*) Explain how missing data were addressed	Page 3 and 5	From the initial 50 AIP cases and 50 controls, there was an insufficient

				amount of urine samples available in the freezer for two of the AIP cases. As a result, these two AIP cases and their corresponding controls were omitted from the urine analysis for the modern kidney and liver biomarkers. Of the 50 AIP cases, 47 of them filled out the diet logbook. The presented dietary data are therefore from 47 AIP cases.
		Case-control study—If applicable, explain how matching of cases and controls was addressed		.
		(*e*) Describe any sensitivity analyses		
**Results**				
Participants	13*	(a) Report numbers of individuals at each stage of study—eg numbers potentially eligible, examined for eligibility, confirmed eligible, included in the study, completing follow-up, and analysed	Page 3 and 5 and Figures 1-11 with descriptions	From the initial 50 AIP cases and 50 controls, there was an insufficient amount of urine samples available in the freezer for two of the AIP cases. As a result, these two AIP cases and their corresponding controls were omitted from the urine analysis for the modern kidney and liver biomarkers. Of the 50 AIP cases, 47 of them filled out the diet logbook. The presented dietary data are therefore from 47 AIP cases
		(b) Give reasons for non-participation at each stage	Page 3 and 5 and Figures 1-11	From the initial 50 AIP cases and 50 controls, there was an insufficient amount of urine samples available in the freezer for two of the AIP cases. As a result, these two AIP cases and their corresponding controls were omitted from the urine analysis for the modern kidney and liver biomarkers. Of the 50 AIP cases, 47 of them filled out the diet logbook. The

				presented dietary data are therefore from 47 AIP cases
		(c) Consider use of a flow diagram		Not performed
Descriptive data	14*	(a) Give characteristics of study participants (eg demographic, clinical, social) and information on exposures and potential confounders	5	The baseline demographic characteristics of the AIP population and the controls were equal on most variables (Table 1).
		(b) Indicate number of participants with missing data for each variable of interest	Page 3 and 5 and Figures 1-11 and Figure A2.	From the initial 50 AIP cases and 50 controls, there was an insufficient amount of urine samples available in the freezer for two of the AIP cases. As a result, these two AIP cases and their corresponding controls were omitted from the urine analysis for the modern kidney and liver biomarkers.<br>Of the 50 AIP cases, 47 of them filled out the diet logbook. The presented dietary data are therefore from 47 AIP cases
Outcome data	15*			
		Case-control study—Report numbers in each exposure category, or summary measures of exposure	Page 6 and Page 10 Page 11	The baseline characteristics of the 50 AIP cases and 50 controls are shown in Table 1. Of the 50 AIP cases, 35 were symptomatic, and 15 were asymptomatic. Of the 48 AIP cases with urine samples for measuring novel kidney markers, 33 were symptomatic AIP cases, and 15 were asymptomatic cases<br>The kidney damage marker FABP-1 in plasma was higher in the group of AIP cases with high urine PBG (n = 30), median 49 pg/mL (IQR = 28-103) compared with the group of AIP cases with low urine PBG (n = 20).<br>A low PBG level was defined as values ≤ 1.5 µmol PBG/mmol

			creatinine, the reference limit for this assay.
Main results 16	(*a*) Give unadjusted estimates and, if applicable, confounder-adjusted estimates and their precision (eg, 95% confidence interval). Make clear which confounders were adjusted for and why they were included	Page 6-16 and page 23, Table 1 and Figures 1-11 and Figure A2.	See Table 1 and Figures 1-11 and Figure A2 (including description)
	(*b*) Report category boundaries when continuous variables were categorized	Page 6-16 and page 23, Table 1 and Figures 1-11 and Figure A2	See Table 1 and Figures 1-11 and Figure A2 (including description)
	(*c*) If relevant, consider translating estimates of relative risk into absolute risk for a meaningful time period		

Continued on next page

Other analyses	17	Report other analyses done—eg analyses of subgroups and interactions, and sensitivity analyses	Appendix Figure A4	Correlation among plasma KIM-1, FABP-1, renin, and α-GST and porphyrins, porphyrin precursors, cytokines and GFR in AIP cases. The figure represents a heatmap of mixed data. The rows represent the different analyses, while the columns represent the individual samples categorized as either AIP cases or controls. The heatmap was generated using single linkage and cosine distance metrics. The expression values are scaled as Z-scores, displayed using a color scale. The figure was generated using Python and was further edited in Adobe Illustrator. The script is available as supplementary information.
**Discussion**				
Key results	18	Summarise key results with reference to study objectives	16	In our study involving AIP patients and matched controls, we analyzed nine novel urine and four plasma markers that detect kidney or liver injury. Based on the known test characteristics of these novel markers characterized in Table A1, our findings indicate that AIP is associated with both proximal tubular kidney damage and hepatocyte damage. Notably, while some of these markers, like KIM-1, have FDA qualification for clinical research, they are not yet approved or available for clinical use. We found no differences in the traditional kidney markers serum creatinine or absolute GFR between AIP cases and matched controls. We have previously reported no

				difference in eGFR between all AIP cases versus matched controls, although differences were observed in subgroups [6]. As for liver markers, serum AST, FIB-4, and ELF were similar in AIP cases versus controls in this study, while a slightly higher serum ALT had previously been observed in AIP cases [6]. Hence, our data suggests that these novel kidney and liver markers, P-KIM-1, P–FABP–1, U-TIMP–1, U-FABP-1, and P-*α-GST, may* detect early-stage damage during a phase of kidney and liver stress before standard kidney and liver markers are elevated
Limitations	19	Discuss limitations of the study, taking into account sources of potential bias or imprecision. Discuss both direction and magnitude of any potential bias	19	This study has several strengths. Firstly, it delivers a unique and comprehensive examination of modern kidney and liver damage markers within the context of AIP, establishing correlations with various biochemical markers. Secondly, the study benefits from a relatively substantial participant cohort, an achievement of significance given the rarity of the disease. Nonetheless, certain limitations warrant consideration. Primarily, the study did not have access to biopsy samples from kidney or ultrasound of liver from the participants which could better verify the data. Furthermore, the study featured a relatively modest sample size while conducting a noteworthy quantity of statistical tests, potentially elevating the risk of encountering false-positive findings. It is pertinent to recognize that most

				participants shared a common pathogenic AIP variant and hailed from Norway. Although this could impact the generalizability of the findings, it is imperative to acknowledge that this pathogenic variant is prevalent in Sweden as well, and all pathogenic AIP variants uniformly manifest as diminished enzyme function
Interpretation	20	Give a cautious overall interpretation of results considering objectives, limitations, multiplicity of analyses, results from similar studies, and other relevant evidence	19	This study assessed biomarkers to identify sensitive and early markers of kidney and liver injury in AIP patients versus matched controls. Elevated plasma levels of the kidney marker KIM-1, the liver marker α-GST and the kidney and liver marker FABP-1 in AIP cases suggest proximal tubular kidney damage and hepatocellular damage. Furthermore, plasma KIM-1 showed a significant positive correlation with the AIP disease activity marker urine PBG and other inflammatory markers like P-CXCL10, CCL2 and TCC. Similarly, in AIP patients, those with high PBG levels had increased FABP-1 in both the plasma and urine. These findings underscore KIM-1 and FABP-1's potential in highlighting subclinical kidney disease in AIP, paving the way for early interventions to curtail kidney damage in AIP
Generalisability	21	Discuss the generalisability (external validity) of the study results		It is pertinent to recognize that most participants shared a common pathogenic AIP variant and hailed from Norway. Although this could impact the generalizability of the findings, it is imperative to

				acknowledge that this pathogenic variant is prevalent in Sweden as well, and all pathogenic AIP variants uniformly manifest as diminished enzyme function.
**Other information**				
Funding	22	Give the source of funding and the role of the funders for the present study and, if applicable, for the original study on which the present article is based	20	Funding: This research was supported by grants from the Somatic Research Fund at Nordland Hospital Trust and the Northern, Norway Regional Health Authorities.

*Give information separately for cases and controls in case-control studies and, if applicable, for exposed and unexposed groups in cohort and cross-sectional studies.

Note: An Explanation and Elaboration article discusses each checklist item and gives methodological background and published examples of transparent reporting. The STROBE checklist is best used in conjunction with this article (freely available on the Web sites of PLoS Medicine at http://www.plosmedicine.org/, Annals of Internal Medicine at http://www.annals.org/, and Epidemiology at http://www.epidem.com/). Information on the STROBE Initiative is available at www.strobe-statement.org.
