# Supplementary material for: Potential Biomarkers for the Earlier Diagnosis of Kidney and Liver Damage in Acute Intermittent Porphyria
Source: Life (Basel). 2023 Dec 21;14(1):19. doi: 10.3390/life14010019 (PMC11154556; doi:10.3390/life14010019)
Supplement: Supplementary file 1 [file life-14-00019-s001.zip › Supplementary Table S2 The code for clustering performed in Figure A8.pdf]

```
# function to plot heatmap
import pandas as pd
import matplotlib.pyplot as plt
from mpl_toolkits.axes_grid1 import make_axes_locatable
import seaborn as sns
from sklearn.preprocessing import StandardScaler

def generate_clustered_heatmap_with_color_options(
        csv_file_path, pdf_file_base_path,
        scaling_method='StandardScaler',
        linkage_method='single',
        distance_metric='cosine',
        color_palette='coolwarm'):

    df = pd.read_csv(csv_file_path, delimiter=',')
    heatmap_data = df.drop(columns=['Case_Control', 'State'])
    heatmap_data.dropna(inplace=True)

    scaler = StandardScaler()
    scaled_data = scaler.fit_transform(heatmap_data)
    scaled_df = pd.DataFrame(scaled_data,
columns=heatmap_data.columns)

    scaled_df_transposed = scaled_df.transpose()

    g = sns.clustermap(
        scaled_df_transposed,
        cmap=color_palette,
        figsize=(30, 12),
        method=linkage_method,
        metric=distance_metric,
        vmin=-6, vmax=6,  # Adjust color scaling
        dendrogram_ratio=(0.09, 0.09),  # Compress both
dendrograms
        cbar_kws={"shrink": 0.5}
    )

    divider = make_axes_locatable(g.ax_heatmap)
    ax_markers = divider.append_axes("top", size="5%",
pad=0.1)

    reordered_indices = g.dendrogram_col.reordered_ind
    reordered_states = df.loc[reordered_indices,
```

```
'State'].values
    ax_markers.set_xlim(0, len(reordered_states))
    ax_markers.axis('off')

    for i, state_value in enumerate(reordered_states):
        if state_value == 0:
            ax_markers.text(i, 0.5, '■', ha='center',
va='center', color='green')
        elif state_value == 1:
            ax_markers.text(i, 0.5, '○', ha='center',
va='center', color='red')
        elif state_value == 2:
            ax_markers.text(i, 0.5, '●', ha='center',
va='center', color='red')

    reordered_case_control = df.loc[reordered_indices,
'Case_Control'].values
    g.ax_heatmap.set_xticks([x + 0.5 for x in
range(len(reordered_case_control))])
    g.ax_heatmap.set_xticklabels(reordered_case_control,
rotation=90, fontsize=8, ha='center')

    legend_labels = ['■: Control (0)', '○: Asymptomatic AIP
(1)', '●: Symptomatic AIP (2)']
    for idx, label in enumerate(legend_labels):
        g.ax_heatmap.text(0, -3 - idx, label, fontsize=10)

    pdf_file_path =
f"{pdf_file_base_path}_{scaling_method}_{linkage_method}_{dis
tance_metric}.pdf"
    g.savefig(pdf_file_path)
    svg_file_path =
f"{pdf_file_base_path}_{scaling_method}_{linkage_method}_{dis
tance_metric}.svg"
    g.savefig(svg_file_path)
csv_file_path = 'elin_data_heat_14_42.csv'
pdf_file_base_path = 'heatmap'  svg_file_path =
'heatmap_cluster4svg'
generate_clustered_heatmap_with_color_options(csv_file_path,
pdf_file_base_path)
```
